# Small Mass but Strong Information: Diagnostic Ions Provide Crucial Clues to Correctly Identify Histone Lysine Modifications

**DOI:** 10.3390/proteomes9020018

**Published:** 2021-04-23

**Authors:** Alaa Hseiky, Marion Crespo, Sylvie Kieffer-Jaquinod, François Fenaille, Delphine Pflieger

**Affiliations:** 1 Studying the Dynamics of Proteins (EDyP), University Grenoble Alpes, CEA, Inserm, IRIG-BGE, 38000 Grenoble, France; alaa.hseiky97@gmail.com (A.H.); marion@adlin-science.com (M.C.); sylvie.kieffer-jaquinod@cea.fr (S.K.-J.); 2 Département Médicaments et Technologies pour la Santé (DMTS), MetaboHUB, Université Paris-Saclay, CEA, INRAE, 91191 Gif sur Yvette, France; francois.fenaille@cea.fr; 3 CNRS, IRIG-BGE, 38000 Grenoble, France

**Keywords:** histones, post-translational modifications (PTMs), sequence variants, proteomic analysis, immonium and diagnostic ions

## Abstract

(1) Background: The proteomic analysis of histones constitutes a delicate task due to the combination of two factors: slight variations in the amino acid sequences of variants and the multiplicity of post-translational modifications (PTMs), particularly those occurring on lysine residues. (2) Methods: To dissect the relationship between both aspects, we carefully evaluated PTM identification on lysine 27 from histone H3 (H3K27) and the artefactual chemical modifications that may lead to erroneous PTM determination. H3K27 is a particularly interesting example because it can bear a range of PTMs and it sits nearby residues 29 and 31 that vary between H3 sequence variants. We discuss how the retention times, neutral losses and immonium/diagnostic ions observed in the MS/MS spectra of peptides bearing modified lysines detectable in the low-mass region might help validate the identification of modified sequences. (3) Results: Diagnostic ions carry key information, thereby avoiding potential mis-identifications due to either isobaric PTM combinations or isobaric amino acid-PTM combinations. This also includes cases where chemical formylation or acetylation of peptide N-termini artefactually occurs during sample processing or simply in the timeframe of LC-MS/MS analysis. Finally, in the very subtle case of positional isomers possibly corresponding to a given mass of lysine modification, the immonium and diagnostic ions may allow the identification of the in vivo structure.

## 1. Introduction

Within a living organism, one cell type is differentiated from another by its own gene expression program. Gene expression is closely controlled by the dynamic modification of DNA-bound histones through the addition of covalent chemical groups on certain amino acids. The most well-known post-translational modifications (PTMs) are methylation and acetylation of histone lysines. Acetylation was discovered in 1963 [1] and numerous studies have progressively established its roles in chromatin accessibility and transcription when it is present at different lysine sites from the core histones H2A, H2B, H3 and H4. Over the past 15 years, further lysine PTMs have been discovered, which likely reveals that the mechanisms of transcription regulation are much more complex. These PTMs are collectively called acylations and differ from acetylation by the length of the side chain (one or two additional CH_2_ groups for propionyl and butyryl, respectively) [2], by the presence of an acidic group such as the carboxylic group (HCO_2_) for malonyl [3] or the ethanoic group (HCO_2_-CH_2_) for succinyl [4]. Further acylations were successively discovered including crotonylation [5], hydroxybutyrylations [6,7], benzoylation [8] and lactylation [9]. Of note, even though these histone PTMs remained “ignored” for decades, it appears that their relative stoichiometry to acetylation is far from negligible on certain lysine residues and in given cellular contexts [6,10].

These lysine acylations were originally discovered by the proteomic analysis of histones digested by the enzyme trypsin. More precisely, the presence of a new acylation on histone peptides was pinpointed by the detection of a mass increment of unknown chemical nature on some peptides. When using MS instruments providing very high mass accuracy (below 5 ppm) and resolution, the precisely measured mass of the modified peptide allowed suggesting only a few possible chemical structures. The nature of the in vivo modified peptide was then determined by comparing its LC retention time and MS/MS fragmentation pattern with those of synthetic peptides bearing the tentative chemical structures of PTMs. This strategy was successfully implemented to validate all newly described acylations on a few histone tryptic peptides [3,4,5,6,7,8,9,10]. Next, the presence of the same mass increment on lysine residues from other histone tryptic peptides was assumed to correspond to the same PTM and led to the establishment of mappings on the sequences of histones H2A, H2B, H3 and H4, indicating that each newly discovered PTM likely modified a large number of histone lysine sites.

Given all these novel lysine PTMs and the existence of histone sequence variants, it becomes a very delicate task to obtain the correct identification of a modified histone peptide, as we previously reviewed in [11]. When focusing on histone H3 and more particularly on its lysine 27 (H3K27), one can observe that many isobaric PTM/variant combinations might exist. A mass difference of two acylations can indeed be equal to the mass difference of two amino acids, especially since H3 can exist in several variants that differ by only a few amino acids. Among these variants, three are well characterized: canonical H3.1 and H3.2 containing the stretch K_27_SAPATGGVK and variant H3.3 exhibiting K_27_SAPSTGGVK. Other genes (*H3mm6-18* and *H3.4/H3t*) encoding H3 variants have been identified in the mouse genome. Many of them appear to be transcribed (in testis, liver, skeletal muscle and/or brain), but only few were identified at the protein level by LC-MS/MS. For instance, *H3mm13* was found transcribed in all four tissues but not formally validated at the protein level [12]; its sequence starting at lysine 27 reads K_27_SVPSTGGVK. One can observe that the mass difference between the sequences from canonical H3 and H3.3 is strictly the mass of an oxygen atom. Besides, the mass difference (V-A) observable between the peptides from H3mm13 and H3.3 is exactly equal to that of a dimethyl moiety. Thus, concluding on the amino acid sequence and the modification state of the H3 peptide analyzed by mass spectrometry can be ambiguous. For instance, acetylation (ac) and propionylation differ by the mass of methyl, acetylation and butyrylation by the mass of dimethyl, and butyrylation (but) and hydroxybutyrylation (ohbu) differ by the mass of an oxygen atom. The previous differences are compensated by the change of amino acids in the three above variants, making the sequences H3mm13-K_ac_SVPSTGGVK, H3.3-K_but_SAPSTGGVK and H3cano-K_ohbu_SAPATGGVK strictly isobaric because they have the same elemental composition, and thus cannot be distinguished even by latest-generation MS instruments offering mass accuracies below 1 ppm. Beside these subtle amino acid variations, chemical artefacts can contribute to the mis-identification of modified histone peptides. Formic acid and acetic acid are indeed often used during protein sample processing and LC-MS/MS analyses. These acids can induce chemical formylation or acetylation of peptide N-termini, which increases the chances of isobaric combinations. For example, acetylation (net addition of H_2_C_2_O) has the same atom composition as the combination of N-terminal formylation (+CO) and methylation (+CH_2_), which can render distinction between lysine acetylation and the sum of N-terminal formylation and lysine methylation challenging.

Former studies have shown the interest of looking in MS/MS spectra for low-mass immonium and diagnostic ions, indicative of the presence of modified lysine residues in the fragmented peptide to ascertain its identification. These ions were scanned, possibly at variable collision energies, to highlight methylated and acetylated sequences [13,14,15]. Tang et al. reported the targeted analysis by Parallel Reaction Monitoring of variably modified peptides from histones H3 and H4; interestingly, by carefully scrutinizing immonium and diagnostic ions, the authors established that H3K27 could be modified by the dual addition of a monomethyl and an acetyl group on its side chain [16]. Propionylation at H3K23 was established in the leukemia cell line U937, notably by verifying the detection of the corresponding diagnostic ion in MS/MS spectra [17]. The usefulness of diagnostic ions to validate numerous lysine PTMs was more systematically assessed in a recent large-scale study [18]. Yet, in the collection of about 5000 synthetic peptides analyzed, less than 1% exhibited an N-terminal modified lysine, whereas such a scheme occurs frequently in histones proteolyzed by trypsin. More recently, a strategy of stepped collisional energy in HCD was suggested to produce both intense diagnostic ions and sequence-determining fragments and allowed identifying sequences containing a few known acylations and pinpointing a new structure in a syntrophic bacterium [19]. Here, we recapitulate the interest of looking for these immonium and diagnostic ions in MS/MS spectra, to validate the identification of peptides from sequence variants of histone H3 bearing a modified lysine at the first position. We carried out this study using histones extracted from mouse testis, because numerous variants are expressed in this organ which are decorated with a wealth of PTMs being dynamically regulated during spermatogenesis [20].

## 2. Materials and Methods

### 2.1. Acid Extraction and Proteolysis by Trypsin of Histones from Whole Mouse Testis

Briefly, whole testes from wild-type mice of C57BL/6 background were ground into fine powder with a mortar and pestle pre-cooled on dry ice. The obtained material was resuspended in 0.2 M sulfuric acid. It was then sonicated using a 3-mm probe CV18 sonicator for 12 cycles of 5 s ON/5 s OFF at 20% amplitude. Lysed cells were incubated on ice for 1 h 30 to extract histones, and then centrifuged at 18,400× *g* for 10 min at 4 °C. The acid extracted histones, contained in the resulting supernatant, were precipitated by incubation with trichloroacetic acid (TCA) on ice for 30 min at a final concentration of 20%. The precipitate was subjected to centrifugation at 18,400× *g* for 15 min at 4 °C. The subsequent histone pellet was washed with cold acetone containing 0.05% HCl, dried under the hood at room temperature, and resuspended in 500 μL of SDS-PAGE loading buffer. The extracted histones were separated on a 12% acrylamide gel. Then, the slice containing H3 was cut and in-gel digested overnight with 0.1 μg trypsin (V511, Promega, Charbonnières, France) using a Freedom EVO150 robotic platform (Tecan Traging AG, Männedorf, Switzerland). Dried tryptic peptides were kept in the freezer until LC-MS/MS analysis.

As an alternative to simple trypsin digestion, we also performed in vitro propionylation of non-modified histone lysines before proteolysis, by following the protocol described in [21] with slight modification. Briefly, the gel bands containing histone H3 were cut into small pieces, destained and finally dried by incubation in pure acetonitrile. The gel pieces were allowed to swell in a solution of 0.1% propionic anhydride in 100 mM TriEthylAmmonium Bicarbonate (TEAB) pH 8.5 and incubated for 10 min at room temperature under agitation at 600 rpm. This step was repeated a second time to increase efficiency of lysine propionylation, and then quenched by addition of 0.09 volume of 80 mM hydroxylamine and incubation for 20 min. Trypsin digestion was then carried out for about 3 h at 37 °C.

### 2.2. LC-MS/MS Analyses of Digested Endogenous Histones and Synthetic Peptides

The synthetic peptides KSAPATGGVK and KSAPSTGGVK bearing different modifications at K1, namely mono-, di- or tri-methylation (me1, me2, me3), acetylation (ac), propionylation (pro) or butyrylation (but), were bought from JPT Peptide Technologies. An equimolar mixture of these synthetic peptides (about 500 fmol each) was analyzed by LC-MS/MS to assess the production of diagnostic ions and measure retention times. To favor in vitro formylation and acetylation of peptide N-termini, this mixture of synthetic peptides was incubated overnight in 5% formic acid or 5% acetic acid, and then diluted to inject about 500 fmol of each peptide for LC-MS/MS analysis. The peptide sequence K_PTM_SAPATGGVK modified with three positional isomers of hydroxybutyrylation, namely 2-hydroxybutyrylation (2ohbu), 3-hydroxybutyrylation (3ohbu) or 2-hydroxyisobutyrylation (hib), was bought from Synpeptide (Shanghai, China). A mixture of 1.5 pmol or 3.2 pmol of each peptide was analyzed on a Q-Exactive HF instrument or on a Q-Exactive+ instrument, respectively (Thermo Fisher Scientific, Illkirch-Graffenstaden, France). Peptides K_PTM_SAPATGGVKme2KPHR, with “PTM” being acetylation, crotonylation (cr) or lactylation (lact), were also bought from Synpeptide. A mixture of 300 fmol or 2 pmol of each was analyzed in four LC-MS/MS runs on a Q-Exactive+ instrument using Normalized Collision Energies (NCE) in HCD of 27%, 30%, 33% or 36%. The three peptides K_PTM_SAPATGGVKme2KPHR were also subjected to in vitro propionylation, following the procedure described above, yet only once for 5 min. An equimolar mixture of the three peptide sequences chemically treated or left otherwise was analyzed on the Q-Exactive+ instrument with stepped collision energies (NCE) at 30%, 33% and 36%, to look in more detail at the efficiency of production of diagnostic ions depending on the propionylation level of the peptide sequences.

All peptide samples were prepared in 2.5% acetonitrile (ACN) and 0.05% trifluoroacetic acid (TFA), and then loaded on a PepMap C18 precolumn (300 μm × 5 mm, Dionex, Thermo Fisher Scientific). They were washed on the precolumn with 0.1% formic acid for 2 min, loaded on and separated by C18 reversed-phase capillary column (75 μm i.d. × 25 cm ReproSil-Pur C18-AQ, 1.9 μm particles) using the UltiMate™ 3000 RSLCnano system (Thermo Fisher Scientific) coupled to a Q-Exactive HF or a Q-Exactive+ mass spectrometer (Thermo Fisher scientific). The capillary column flow rate was around 300 nL/min. The mobile phases consisted of solution A (water with 0.1% formic acid) and solution B (acetonitrile with 0.08% (*v/v*) formic acid). Peptides were eluted with a gradient consisting of an increase in solvent B from 2% to 7.5% in 5 min, then from 7.5% to 33.2% over 33.5 min, from 33.2% to 49% over 6.5 min, followed by a 13-min flush of the column at 72% B.

Mass spectrometry acquisitions were of two types. Exploratory analyses, also named Data-Dependent Acquisitions, aimed at fragmenting as many peptides as possible and were used for several synthetic peptide samples and for the endogenous histones. These acquisitions were carried out by alternating one full MS scan with Orbitrap detection acquired over the mass range 300 to 1300 *m/z*, at a target resolution of 60,000, with a maximum injection time of 100 ms and an AGC of 10^6^, and data-dependent MS/MS spectra on the 10 most abundant precursor ions detected in MS. The peptides were isolated for fragmentation by higher-energy collisional dissociation (HCD) with a normalized collision energy of 27% (except otherwise stated in the text) using an isolation window of 2 *m/z*, a target resolution of 60,000, a maximum injection time of 250 ms and AGC of 10^6^. MS/MS spectra were recorded starting at *m/z* 80 to detect all types of diagnostic ions for non-modified and modified lysine residues. Dynamic exclusion of already fragmented species was applied for 30 s. Besides, targeted analyses consisted of accumulating many spectra on some peptides of interest. They thus provided very good statistics on the relative intensities of the produced fragments. Such analyses were performed on the synthetic hydroxybutyrylated peptides K_PTM_SAPATGGVK, by specifying the fragmentation of the mass to charge ratio 501.2854 (2+).

### 2.3. Interpretation of Proteomics Data

After peptide analysis by LC-MS/MS, the obtained RAW files were converted into MGF files using the program Mascot Distiller from Matrix Science (http://www.matrixscience.com/, accessed on 23 April 2021). We selected the option of “S/N” for ion intensities, which allowed producing MGF files and thus spectra interpreted by Mascot with relative fragment intensities reflecting quite well those observable in the original MS/MS spectra. By contrast, the option “area” tends to reduce detection of low-mass fragments. Then, MS/MS data were interpreted against a mouse-histone-only database developed in our group [22]; the precursor mass tolerance was 5 ppm while the fragment mass tolerance was 25 mmu; enzyme specificity was trypsin with a maximum of five missed cleavages allowed. We were interested in studying PTMs on lysine residues including methylation, dimethylation, trimethylation, acetylation, propionylation, butyrylation, crotonylation, hydroxybutyrylation and lactylation, in addition to N-terminal acetylation or N-terminal formylation of peptides, and thus specified them as variable PTMs in Mascot. The list of variable PTMs was adapted to the question asked, so as not to exceed a total of nine PTMs specified for each conducted database search.

## 3. Results

### 3.1. Diagnostic Ions and Neutral Losses Reveal the Nature of PTMs on Lysine Residues of Fragmented Peptides

**Figure 1:**
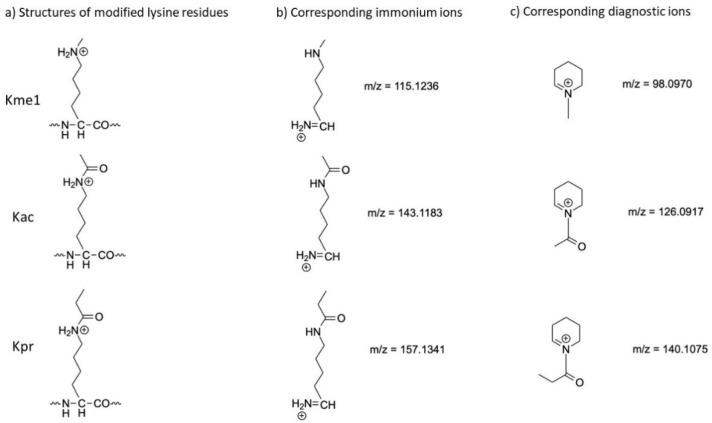
(**a**) Proposed structures of lysine residues modified by me1, ac or pro, and of their corresponding (**b**) immonium and (**c**) immonium-related diagnostic ions.

A lysine residue, possibly bearing a modification within a peptide, produces an immonium ion during MS/MS fragmentation. This immonium ion can further lose ammonia (α-NH3) to generate a diagnostic ion, a 6-membered ring (cycle) that is very stable (Figure 1). Diagnostic ions are highly specific for each type of modified lysine and thus increase the confidence of lysine PTM identification.

**Figure 2:**
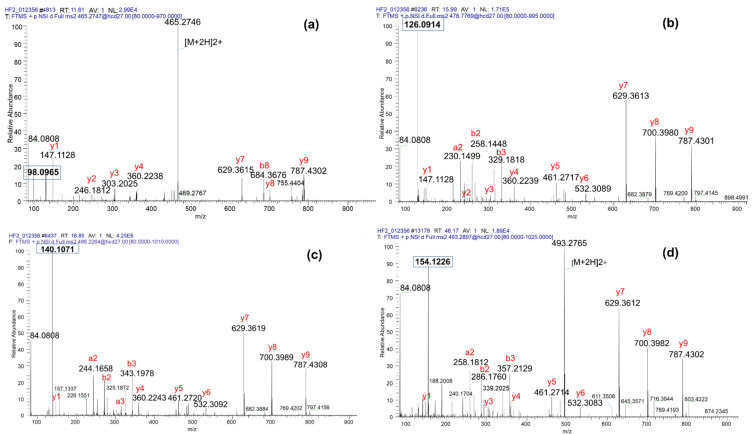
HCD MS/MS spectra of variably modified K_PTM_SAPATGGVK synthetic peptides in their doubly charged state. (**a**) Methylated peptide with detection of the diagnostic ion for K_me1_ at 98.097. (**b**) Acetylated peptide with detection of the diagnostic ion for K_ac_ at 126.091. (**c**) Propionylated peptide with detection of the diagnostic ion for K_pro_ at 140.107. (**d**) Butyrylated peptide with detection of the diagnostic ion for K_but_ at 154.123.

We listed in Table 1 the theoretical masses of diagnostic and immonium ions of lysine residues exhibiting several PTMs. We analyzed by LC-MS/MS synthetic peptides H3cano-K_27_SAPATGGVK and H3.3-K_27_SAPSTGGVK bearing different modifications on the N-terminal lysine, and assessed the extent of detection of the corresponding immonium and diagnostic ions. One can note that in the case of an N-terminal modified lysine, the immonium ion corresponds to fragment a1 and the diagnostic ion to a1-NH_3_. We will nonetheless use the general naming of diagnostic and immonium ions to be able to address the cases of peptides containing several modified lysine residues. We observed that from the peptides bearing methyl (Figure 2a), acetyl (Figure 2b), propionyl (Figure 2c) or butyryl (Figure 2d), we could detect intense fragments with *m/z* ratios at 98.096, 126.091, 140.107 and 154.123, respectively, diagnostic ions that allowed revealing the nature of the PTM present in the peptide. Modifications of lysine by me2 or me3 constitute di-substitutions on the lateral chain. Upon MS/MS fragmentation of peptides containing Kme2 or Kme3, a fragment at *m/z* 130.09 is observed which corresponds to protonated pipecolic acid, generated following losses of dimethylamine or trimethylamine, respectively [23,24] (Supplementary Material Figure S1a,b). This ion is formed in addition to the lysine nominal acylium ion at *m/z* 129.10 [24]. Further inspection of the spectra acquired on the trimethylated peptides indicated a neutral loss of 59.07 from b2 and b3 fragments, as well as from the doubly charged precursor (Figure S1b). The loss of 59.07 was already described as the loss of trimethylamine [N(CH_3_)_3_] from b2 fragment upon CID fragmentation and to be specific to trimethylated peptides [25]; it is actually taken into account by the program Mascot for MS/MS data interpretation. In the case of K_me2_, a loss of 45.06 representing dimethylamine (NH(CH_3_)_2_) could be observed upon the fragmentation of dimethylated lysine amino acids [26]. Yet, this loss was not observed in our analyses of K_me2_-containing peptides (Figure S1a). This loss has rather been attributed to peptides containing an asymmetrically dimethylated arginine [27]. In conclusion, upon the fragmentation of the short sequence KSAP(A/S)TGGVK modified with monomethylation, acetylation, propionylation and butyrylation on the first lysine (mono-substitutions), characteristic diagnostic ions of Kmod were readily produced when using an HCD normalized collision energy (NCE) of 27%.

### 3.2. Formylation or Acetylation at the N-Terminal of Peptides Does Not Prevent Production of the Diagnostic ion of K1_PTM_

The proteomic analysis of histones is often performed by including a step of in vitro propionylation of lysine residues before and after trypsin digestion: non-modified lysines (and to a large extent monomethylated lysines) and peptide N-termini thus become propionylated. On the one hand, this allows reducing the number of trypsin cleavage sites, thus obtaining longer and more hydrophobic peptides which are better retained on the C18 column, and this increases their ionization efficiency. On the other hand, this treatment simplifies quantitative analyses because the same amino acid sequence ending with Arg is formed whatever the modification status of the lysines. When one’s research interest is to study all the modifications on histone lysines including propionylation, in vitro propionylation is often not performed. This makes the N-terminus of the peptides exposed to chemical modification and increases the chances of occurrence of strictly isobaric PTM combinations (Table 2). Indeed, we use a 5% formic acid solution to extract proteolytic peptides from gel bands, and this acid is present in the LC buffers so that the chemical formylation of peptide N-termini can be expected. Alternatively, acetic acid may be used during sample processing, possibly leading to N-terminal acetylation.

To test whether N-terminal formylation or acetylation might hamper the production of diagnostic ions, we incubated overnight in 5% formic acid or 5% acetic acid the mixture of 12 synthetic peptides of sequences K_PTM_SAPATGGVK and K_PTM_SAPSTGGVK, with “PTM” being me1, me2, me3, ac, pro and but. LC-MS/MS analysis of the resulting sample was followed by data interpretation with Mascot, by including N-terminal formylation or acetylation as possible modifications.

The program identified the N-terminally formylated sequences bearing a propionylation or a butyrylation on K1. Supplementary Material Figure S2a,b represent the MS/MS spectra assigned to fo-K_pro_SAPATGGVK and fo-K_but_SAPATGGVK. Diagnostic ions were indeed detected in the low mass range at *m/z* 140.107 and 154.123, respectively, as expected for these two lysine PTMs. Mascot also suggested the identification of fo-K_me3_SAPATGGVK from certain MS/MS spectra. The detection of an intense fragment at 154.123 allowed correcting the identification of the fragmented peptide as being K_but_SAPATGGVK, which is of strictly the same mass as fo-K_me3_SAPATGGVK (Supplementary Material Figure S2c). The identification of K_but_SAPATGGVK was properly proposed by Mascot, with a very close score, when the possible N-terminal formylation was removed from the search criteria. Of note, fo-K_pro_SAPATGGVK and fo-K_but_SAPATGGVK were also identified when the peptide mixture was simply in contact with 0.1% formic acid during LC-MS/MS analysis, which further strengthens the interest of examining the low mass range of MS/MS spectra to validate peptide identifications.

The sequences ac-K_ac_SAPSTGGVK (Supplementary Material Figure S2d), ac-K_pro_SAPSTGGVK (Supplementary Material Figure S2e) and ac-K_but_SAPSTGGVK (Supplementary Material Figure S2f) were detected with their diagnostic ions at 126.091, 140.107 and 154.123, respectively. Similar observations were made on the sequence KSAPATGGVK from canonical histone H3. In some instances, Mascot suggested the identification of the sequence ac-K_pro_SAPSTGGVK but without the diagnostic ion of K_pro_: instead, the spectrum contained the diagnostic ion of K_but_ at 154.12, indicating that the correct identification was the alternative sequence of the very same mass, fo-K_but_SAPSTGGVK (Supplementary Material Figure S2g).

Regarding peptide sequences variably methylated on K1, we did not identify their N-terminally formylated or acetylated forms, even when scrutinizing the MS/MS data visually. It might be that these modifications prevent the chemical modification of the peptide N-terminus, whereas propionylation and butyrylation allow them up to a few percent, as estimated by the MS signals of the N-terminally modified versus non-modified peptides. Of note, the relative intensity of the diagnostic ions for Kpro and Kbut was important, being close to the base peak in many spectra (Supplementary Material Figure S2a,b,d–f). Therefore, the chemical modification of the lysine N-terminus by formic or acetic acid does not affect the production of the diagnostic ion, which reveals the nature of the PTM born by lysine K1 in the fragmented peptide.

### 3.3. Usefulness of the Immonium and Diagnostic Ions to Discriminate Positional Isomers of Lysine Hydroxybutyrylation

Hydroxybutyrylation is a PTM that can exist in five positional isomers: all contain an OH group differently anchored on a linear or branched butyrylation [6,7]. Among the possible isomers, 2-hydroxyisobutyryl (hib) was identified as a histone mark in mouse testis and in human Hela cells. As many as 63 K_hib_ sites were mapped on human and mice histones, including on H3K27. Interestingly, among these modified sites, 27 were unique to K_hib_ and were not subjected to acetylation [6]. Besides, β-hydroxybutyrylation (or 3-hydroxybutyrylation) was identified by LC-MS/MS analysis and described to modify histones, with 44 histone K_3ohbu_ sites identified in liver cells obtained from mice subjected to prolonged fasting [7]. H3K9, H3K4 and H4K8 were among these sites; H3K27_3ohbu_ was not detected in mice liver histones, yet it was identified in human cells.

We could obtain the synthesis of K_PTM_SAPATGGVK peptide bearing either 2-hydroxybutyrylation (2ohbu), 3-hydroxybutyrylation (3ohbu), or 2-hydroxyisobutyrylation (hib). The peptides bearing these variable PTMs were expected to all produce the same immonium and diagnostic ions at *m/z* 187.144 and 170.118, respectively, upon MS/MS fragmentation. Of note, the PTM 4ohbu was described to be discriminated from other isomers by the neutral loss of 86.03 from the precursor ion: such a loss was indeed detected upon the fragmentation of several peptides, including DAVTYTEHAK_4ohbu_R, VTIMPK_4ohbu_DIQLAR, and K_4ohbu_TVTAMDVVYALK [6].

We observed that the three synthetic peptides had barely different chromatographic retention times when injected separately: 17.40 min for 3ohbu, 18.05 min for hib and 18.17 min for 2ohbu. When the three isomers were injected together, the retention times were a bit different (16.91, 17.49 and 18.01 min) (Supplementary Material Figure S3a). This retention time change may be explained by run-to-run variability or by a “matrix effect”, which is the effect of the other co-analyzed peptides on the chromatographic behavior of a considered peptide. Determining the identity of an endogenous peptide K_ohbu_SAPATGGVK based on retention times would require well-thought successive additions of synthetic peptides to the endogenous sample [6], or the use of synthetic peptides containing ^13^C/^15^N-coded residues, to induce a mass shift as compared to the endogenous species [9].

**Figure 3:**
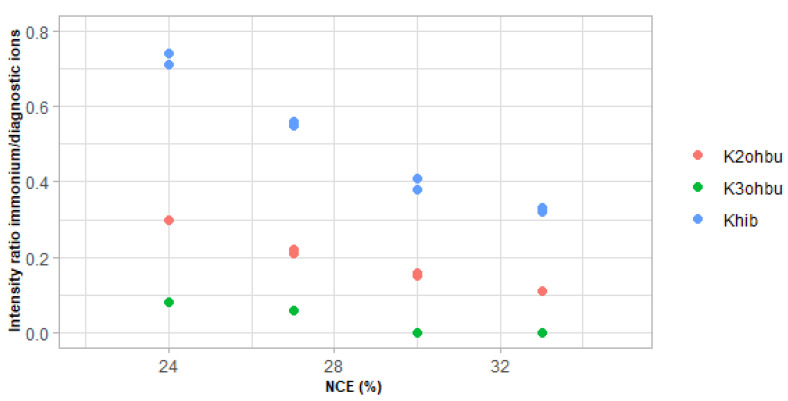
Variation of the intensity ratio between the immonium and the diagnostic ions of Kohbu, at *m/z* = 187.144 and 170.118, respectively, with the Normalized Collision Energy (NCE) used for MS/MS fragmentation of the synthetic peptides K_ohbu_SAPATGGVK. LC-MS/MS analyses were performed in duplicate at each NCE.

**Figure 4:**
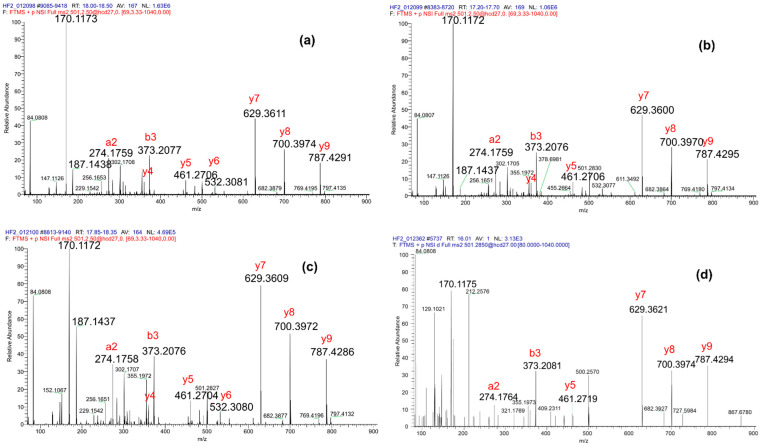
MS/MS spectra acquired on K_PTM_SAPATGGVK with PTM being various positional isomers of hydroxybutyrylation. Sum over 0.5 min of MS/MS scans acquired on the three synthetic peptides (**a**) K_2ohbu_SAPATGGVK, (**b**) K_3ohbu_SAPATGGVK and (**c**) K_hib_SAPATGGVK. (**d**) MS/MS spectrum (single scan) acquired on a tryptic peptide from histone H3 extracted from a cell suspension of mouse testis.

Alternatively, we wondered whether diagnostic and immonium ions might contribute to distinguishing peptides modified with various isomers of lysine hydroxybutyrylation. We performed the targeted MS/MS analysis of the sequence K_PTM_SAPATGGVK, with PTM being 2ohbu, 3ohbu and hib, at NCE values of 24%, 27%, 30% and 33% using a Q-Exactive+ instrument. Summing spectra over the chromatographic peak of each peptide revealed near identity of fragmentation patterns in terms of b and y fragments. By contrast, the relative intensity of the immonium and diagnostic ions varied substantially between peptides bearing the various hydroxybutyryl isomers (Figure 3). The analysis of the same mixture of three peptides on a Q-Exactive HF instrument at NCE 27% provided very similar results, with fragmentation patterns mirroring each other, except for diagnostic/immonium intensity ratios, which were equal to 5 for K_3ohbu_, 15 for K_2ohbu_ and 55 for K_hib_ (Figure 4a–c). Therefore, the relative intensity between the immonium and diagnostic ions is a feature that differentiates the peptides K_PTM_SAPATGGVK bearing the various tested hydroxybutyryl isomers.

The LC-MS/MS analysis of tryptic peptides from histone H3, acid-extracted from a cell suspension from mouse testis, presumably identified K_ohbu_SAPATGGVK from an MS/MS spectrum similar to those of the above synthetic peptides, and at a retention time around 16.0 min (Figure 4d). The fragment at *m/z* = 170.118 was the second peak of maximum intensity and probably corresponded to the diagnostic ion of K_ohbu_; by contrast, no immonium ion was detected at *m/z* = 187.144. This indicates that the N-terminal lysine is probably modified by 3ohbu. This peptide was rarely identified in our histone samples extracted from whole mouse testis, presumably because it is very hydrophilic and possesses poor ionization efficiency. Yet, the fragmentation pattern of the endogenous species is very close to the MS/MS spectrum acquired at the beginning of the chromatographic peak of the synthetic peptide K_3ohbu_SAPATGGVK (Supplementary Material Figure S3b). In conclusion, it is very probable that the peptide K_ohbu_SAPATGGVK detected in histone H3 purified from whole mouse testis is modified by 3-hydroxybutyrylation, also named beta-hydroxybutyrylation.

### 3.4. Adjusting Normalized Collision Energy (NCE) Values on the Longer Peptide K_PTM_SAPATGGVK_me2_KPHR

In a previous study exploring histone lysine crotonylation in the context of mouse spermatogenesis, we have shown that H3K27 is modified by this acylation at a similar level to acetylation in the latest stages of the differentiation process. More precisely, H3K27cr was detected in combination with either H3K36unmod or H3K36me2 [10]. In that work, we did not optimize MS analysis conditions to produce the diagnostic/immonium ions of Kcr; of note, its structure containing an unsaturation dramatically reduces possibilities of isobaric PTM combinations. Here, we wished to test whether NCE needs to be adjusted to produce both sequence-informative y/b fragments and the diagnostic ion corresponding to K_PTM_ from the peptide K_PTM_SAPATGGVK_me2_KPHR. We considered acetylation as a reference PTM, and crotonylation and lactylation as much less explored PTMs. An equimolar mixture of the three peptides was analyzed by successive LC-MS/MS runs using NCE values of 27%, 30%, 33% and 36%. The three peptides were most often selected for fragmentation in their quadruply charged form. The MS/MS spectra corresponding to the analysis of 300 fmol of each peptide and leading to highest score identifications by the program Mascot are shown in Supplementary Material Figure S4a–f. For the three sequences, the NCE of 27% provided identification of the lowest scores, indicating that sub-optimal fragmentation of the peptide backbone was obtained. NCE values of 30% and 33% provided balanced intensities between y/b fragments and diagnostic ions, detected at 126.092, 152.107 and 156.102, respectively. In addition, one or the other of these two NCE values provided optimal coverage of the peptide N-terminus in terms of b2 and b3 fragments, which is interesting to discriminate between canonical H3 and H3.3 on the one hand, and H3mm13 on the other hand. The analysis of 2 pmol of each peptide led to similar observations. In conclusion, when analyzing the sequence K_PTM_SAPATGGVK_me2_KPHR or similar ones varying in terms of H3 variants and of PTMs present at K36 and/or K37, it can be worth designing a method that combines the use of two or three NCE values between 30% and 36% (stepped collision energy) to obtain both reliable sequence determination and intense detection of diagnostic ions.

### 3.5. When In Vitro Propionylation Is Performed before Trypsin Digestion

We already mentioned that the discovery of new lysine acylations was most often obtained by the LC-MS/MS analysis of histone samples simply digested by the enzyme trypsin. Indeed, in vitro propionylation precludes the analysis of the endogenous form of this PTM; it also masks endogenous lysine butyrylations because monomethylated lysines can additionally become propionylated. Finally, in vitro propionylation may lead to side-reactions on other amino acids [28,29,30]. Yet, to perform the comparative analysis of histone samples, the chemical propionylation of non-modified lysine residues before histone proteolysis is advantageous, without necessarily including the step of the modification of tryptic peptide N-termini that aims at further increasing ionization efficiency [21]. We wished to assess how this chemical modification before trypsin digestion would impact the production of diagnostic ions from H3K27_PTM_ during MS/MS analysis of the sequence spanning K27–R40 from histone H3. We then analyzed histone H3 acid-extracted from a cell suspension from whole testis, in vitro propionylated before trypsin digestion. A set of modified sequences K27–R40 identified when using an NCE of 27% is shown in Table 3.

The sequence K27–R40 doubly propionylated at H3K36 and H3K37 was identified with the PTMs me1, me2, me3 and ac on H3K27. In all these cases, the diagnostic ion corresponding to K_pro_ was detected with high intensity, above 75% of the intensity of the dominant peak (base peak) in the MS/MS spectrum. When H3K36 bore another PTM, the diagnostic ion for K_pro_ dropped substantially. Mono- and tri-methylation on H3K27 were identified with the production of the expected diagnostic ion or neutral losses. The diagnostic ion for K_me1_ was not very intense; this is in line with the trend observed on the variably modified short peptides K_PTM_SAPATGGVK, for which the lowest intensity diagnostic ion was observed when “PTM” was methylation (Figure 2). Acetylation on H3K27 was detected in three H3 variants, namely canonical H3, H3.3 and H3mm13. In the latter sequence variant, the diagnostic ion for K_ac_ was of low intensity, though. The existence of H3mm13 at the protein level was better confirmed with the identification of K_ac_SVPSTGGVK_me2_K_pro_PHR, which produced diagnostic ions for K_ac_ and K_pro_ of similar intensities, and of K_pro_SVPSTGGVK_pro_K_pro_PHR, which allowed the detection of fragments a1 and a2 in addition to an intense diagnostic ion for K_pro_, thus validating the valine at position 3 in the peptide. To our knowledge, this is the first report of variant H3mm13 being present at the protein level in mouse testis by the detection of several modified forms of the peptide K27SVPSTGGVKKPHR. Finally, the interpretation of the MS/MS data by including butyrylation as a possible lysine PTM brought a few tentative identifications within H3.3. We could rule them out and correct them for being the double modification by me1 and pro by the detection of the diagnostic ions for both PTMs at 98.097 and 140.107. When performing an LC-MS/MS analysis using stepped normalized collision energy at 30% and 33%, we made similar observations. In conclusion, the propionylation of H3K36 and H3K37 usually leads to the production of an intense diagnostic ion for Kpro, and seems to impact to variable extents the production of the diagnostic ions for K27_PTM_, depending on the H3 sequence variant and the nature of K27_PTM_.

### 3.6. Testing the Production of Diagnostic Ions for K27_PTM_ from Synthetic Peptides K_PTM_SAPATGGVK_me2_KPHR with Variable Levels of In Vitro Propionylation

We have observed above that the propionylation of H3K36/K37 may reduce the production of the diagnostic ion for H3K27_PTM_. We then wished to compare more precisely the behavior of the synthetic peptides K_PTM_SAPATGGVK_me2_KPHR (PTM being ac, cr or lact) with no chemical treatment, with complete propionylation of K37 and the peptide N-terminus and with partial propionylation (only at H3K37 or at the peptide N-terminus). An equimolar mixture of the three peptide sequences in vitro propionylated or non-treated was analyzed on the Q-Exactive+ instrument. Based on our former analyses of the three non-propionylated peptides, we implemented an analysis using stepped collision energies (NCE) at 30%, 33% and 36%.

**Figure 5:**
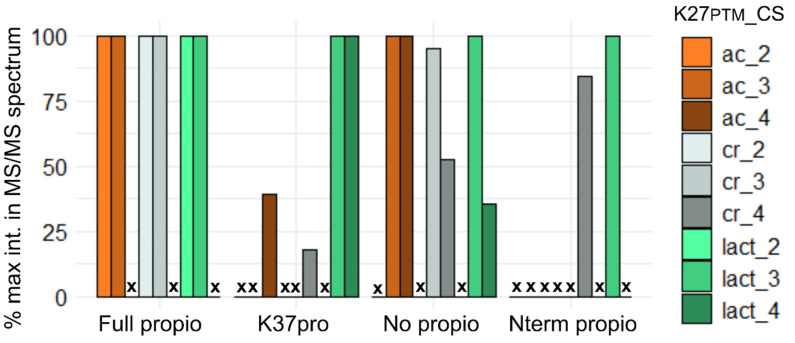
Efficiency of production of the diagnostic ion for K27_PTM_ within sequence K27_PTM_SAPATGGVKme2KPHR, when PMT is ac, cr or lact and the peptide is variably propionylated. The percentage of intensity of the diagnostic ion in the MS/MS spectrum leading to peptide identification has been plotted in function of the peptide propionylation level (fully propionylated, only modified at K37, not propionylated, or only thus modified at its N-terminus). The nature of the PTM and the charge state (CS) of the precursor ion have been taken into account as variables. Crosses (x) indicate that no identification has been obtained in the considered states (propionylation state, PTM nature, CS).

We observed that the incomplete reactions of propionylation occurred to a minor yet sufficient extent to obtain identifiable MS/MS spectra. Figure 5 represents the relative intensities of the diagnostic ion coming from K27_PTM_ in the MS/MS spectra (value is 100 if the diagnostic ion is the base peak). One can observe that for the three PTMs considered, the diagnostic ion of K27_PTM_ was the most intense peak in the MS/MS spectra acquired on the fully propionylated peptides. In contrast, the relative intensity of this diagnostic ion varied with the nature of the PTM born by H3K27 and the peptide charge state (CS) when no propionylation was performed or when only H3K37 was thus modified. One can also note that full propionylation modified the CS of ionized peptides providing identifications: from 3+ and 4+ in the absence of N-terminal modification to 2+ and 3+ when the N-terminus was propionylated. Finally, the study on this small set of peptides supports carrying out full peptide propionylation (of free and monomethylated lysine residues before tryptic digestion and of the N-terminus of resulting peptides), in combination with a strategy of MS/MS fragmentation using stepped collision energy to obtain both peptide identification and intense diagnostic ions confirming the identity of K27_PTM_.

### 3.7. Can Retention Times Be Useful to Differentiate Variably Modified K_PTM_S(A/V)P(A/S)TGGVK?

Besides the detection of diagnostic ions, retention times can be scrutinized to determine the nature of the modification. Indeed, modified peptides can be expected to elute differently depending on the hydrophilic or hydrophobic nature of their PTMs. The peptides K_PTM_SAPATGGVK and K_PTM_SAPSTGGVK bearing me1, me2, me3, ac, pro and but analyzed above eluted at retention times (RT) 11.0, 11.15, 11.15, 15.4, 16.7 and 18.8 min, respectively; methylated peptides gave rise to substantial peak tailing. Such RT values are in concordance with the study performed on a large collection of tryptic peptides containing modified lysines, which stated that variably methylated sequences did not become separated, while acylated peptides had increasing RT with an increasing number of carbon atoms in the PTM structure [18]. In the case of the sequence K27–R40 from histone H3, two other parameters influence RT. First, the variations in amino acids at position 29 and 31 may impact RT as much as a change of lysine PTM. Besides, proline residues can exist in two configurations, cis and trans, which lead to different RT. Whereas the vast majority of prolines exist in the trans configuration within proteins, proline isomerization may occur enzymatically. It can also happen spontaneously after trypsin digestion. When analyzing histone peptides specific of H2A or H2B sequence variants and containing one or several proline residues, we indeed observed the appearance of (at least) two chromatographic peaks separated by several minutes, upon successive sample injections separated by freeze–thaw cycles [31]. When looking carefully at the MS/MS data acquired on a tryptic digest from histone H3 obtained from mouse male germ cells, we observed that the sequence KacSAPSTGGVKme2KPHR had probably been identified at two distinct retention times (Supplementary Materials Figure S5a–c). One likely explanation is that the chromatographic peak of minor abundance and eluting earlier corresponds to the peptide sequence containing (at least) one proline in cis configuration. In conclusion, when analyzing variably acylated peptides containing a proline and/or subject to possible sequence variations, retention times do not constitute a robust indication of the lysine PTM.

## 4. Discussion

A wealth of PTMs have been described over the past decade to possibly modify histone lysines, which renders the task of correctly identifying the combination of peptide sequence and PTMs very challenging. In this work, we wanted to assess whether some analytical features could help ascertain the identity of modified histone peptides. We focused on the peptide of histone H3 starting at lysine 27 for several reasons. First, this residue is known to bear several PTMs, including H3K27me3 and H3K27ac, but also various other acylations. Second, residues 29 and 31 differ between H3 variants and these changes match the masses of PTMs (Ala ≥ Val matches me2 or a switch from ac and buty; Ala ≥ Ser matches a switch from buty to ohbu). As the produced MS/MS fragments do not necessarily cover the 3 to 5 N-terminal amino acids, the modification status of the peptide N-terminal lysine can be ambiguous. Our study highlights that the low-mass diagnostic ions, which are often given little attention because they do not inform on the amino acid order in the analyzed peptide sequence, can actually provide valuable information. To obtain more reliable identification and mapping of PTMs, we recommend the examination of these low mass ions, as well as checking for neutral losses. We observed that N-terminal formylation or acetylation can occur just in the timeframe of the LC-MS/MS analysis, and render the identification of peptides bearing in vivo PTMs even more challenging. Yet, we verified that these N-terminal chemical modifications did not prevent the production of the diagnostic ions during MS/MS. When in vitro propionylation of histones is considered, it should include the step of N-terminal modification of tryptic peptides, to favor the production of the diagnostic ion of the first lysine residue. The use of other chemicals to modify peptide N-termini [21] could be further tested for their effect on the formation of this diagnostic ion. Finally, we verified again that an MS acquisition method including stepped collision energy favors the production of both sequence-informative fragment and diagnostic ions. Of course, the usefulness of diagnostic ions is only true when the co-fragmentation of two peptides has been avoided.

In this work, we used non-resonant Higher-Energy Collisional Dissociation (HCD) as the fragmentation technique, which allows the efficient detection of the diagnostic ions in the low mass region. In former proteomics studies, carried out on histones or other protein samples, the other fragmentation technique resonant Collision Induced Dissociation (CID) has been heavily used for its acquisition speed and sensitivity. However, when fragmenting a peptide under resonant conditions, the fragments of mass to charge (*m/z*) ratios below 1/3 of the *m/z* of the fragmented peptide are not stable (low mass cut-off) and thus not detected in the final MS/MS spectrum. In conclusion, the diagnostic ions were often not accessible in these CID MS/MS spectra. We could, however, verify in the studies reporting the discovery of hydroxybutyrylated lysines that the diagnostic ion at 170.11 could be detected in some spectra, which supported the nature of the lysine PTM in the analyzed peptides. With the use of HCD, and more generally of fragmentation techniques giving access to the low-mass range with high mass accuracy, it may be advisable to pay more attention to immonium/diagnostic ions. Beyond the peptide of histone H3 starting at H3K27, many tryptic peptides from H3 and other histone sequences can start with a modified lysine (e.g., H3-K9STGGKAPR, H3-K18QLATKAAR, etc.), some of which can again include variations of amino acids (e.g., H3-K18QLATKAAR versus TSH3-K18QLATKVAR). Beyond histone analysis, these small-mass ions may be useful in large-scale analyses of tryptic peptides bearing acylated lysines, enriched by affinity-purification using antibodies that possibly exhibit cross-reactivity with closely related chemical structures.

In conclusion, our work demonstrates further that the identification of modified histone peptides can often be ambiguous and requires very careful analysis, including the close visual inspection of the MS/MS spectra in terms of low-mass diagnostic ions and of neutral losses.

## Figures and Tables

**Table 1:** List of the theoretical masses of diagnostic and immonium ions produced from lysine residues bearing various PTMs.

Lysine Modification	M (Diagnostic Ion)	M (Immonium Ion)
non-modified	84.0813	101.1079
methyl	98.0970	115.1236
formyl	112.0757	129.1023
acetyl	126.0917	143.1183
propionyl	140.1075	157.1341
butyryl	154.1226	171.1492
crotonyl	152.1070	169.1336
hydroxybutyryl	170.1176	187.1442
lactyl	156.1019	173.1285

**Table 2:** This table lists a series of PTMs and PTM combinations that are of strictly same mass.

Mass	PTM	Isobaric PTM Combination 1	Isobaric PTM Combination 2	Isobaric PTM Combination 3
42.0106	acetyl	formyl + methyl	--	--
56.0262	propionyl	acetyl + methyl	formyl + dimethyl	--
70.0419	butyryl	propionyl + methyl	formyl + trimethyl	acetyl + dimethyl
100.0160	succinyl	malonyl + methyl		
114.0317	glutaryl	hydroxybutyryl + formyl	malonyl + dimethyl	succinyl + methyl

**Table 3:** Peptide sequences spanning residues K27–R40 from canonical H3, variant H3.3 and H3mm13 identified in mouse testis with various sets of PTMs. Detection of the diagnostic ions or neutral losses corresponding to K_PTM_ was verified in the original MS/MS spectra using QualBrowser. The NCE was 27% in this analysis. CS_prec_ designates the charge state of the precursor ion selected for fragmentation. Int (diag) is the intensity of the diagnostic ions for either N-terminal K27 or propionylated K36/K37; we set to 100 the one of higher intensity for comparing both. “%max int (diagK_pro_)” positions the diagnostic ion of K_pro_ as compared to the fragment of highest intensity in the MS/MS spectrum.

Histone	Probable Identified Sequence	CS_prec_	Int (diagK27_PTM_)	Int (diagK_pro_)	Int (diagK27_PTM_)/Int (diagK_pro_)	%Max Int (diagKpro)
Cano H3	K_me1_SAPATGGVK_pro_K_pro_PHR	3 or 4	10.4	100	0.10	100
Cano H3	K_me3_SAPATGGVK_pro_K_pro_PHR	3 or 4	N.A. ^(a)^			90 or 75
H3.3	K_me3_SAPSTGGVK_pro_K_pro_PHR	3	N.A. ^(a)^			95
Cano H3	K_ac_SAPATGGVK_pro_K_pro_PHR	3	100	75	1.25	75
H3.3	K_ac_SAPSTGGVK_pro_K_pro_PHR	2	100	31	3.2	25
H3mm13	K_ac_SVPSTGGVK_pro_K_pro_PHR	3	3.1	100	0.03	97
H3mm13	K_ac_SVPSTGGVK_me2_K_pro_PHR	4	95	100	0.95	10
H3mm13	K_pro_SVPSTGGVK_pro_K_pro_PHR	3	N.A.			100
H3.3	K_(me1pro)_SAPSTGGVK_pro_K_pro_PHR	3	23	100	0.23	100
H3.3	K_(me1pro)_SAPSTGGVK_(me1pro)_K_pro_PHR	2	100	80	1.25	12

^(a)^ K_me3_ does not produce a diagnostic ion, yet we always verified the loss of trimethylamine (−59) from fragment b3, and possibly from b3-H_2_O, b2 or a2.

## Supplementary Materials

The following are available online at https://www.mdpi.com/article/10.3390/proteomes9020018/s1, Figure S1: MS/MS spectra of K_PTM_SAPATGGVK exhibiting diagnostic ions, neutral losses or no specific feature indicative of the peptide PTM; Figure S2: MS/MS spectra acquired on synthetic acylated peptides K_PTM_SAPATGGVK or K_PTM_SAPSTGGVK additionally modified by an N-terminal formylation or acetylation.; Figure S3: analysis of synthetic hydroxybutyrylated peptides of sequence KhbSAPATGGVK.; Figure S4: Optimization of the normalized collision energy (NCE) used for the MS/MS analysis of the synthetic acylated peptides K_PTM_SAPATGGVKme2KPHR; Figure S5: identification of the peptide K27acSAPSTGGVKme2KPHR in two separate chromatographic peaks during the analysis of endogenous histone H3 digested by trypsin, thus probably corresponding to the same sequence varying by the presence of proline(s) in trans *versus* cis configuration.
