# Supplementary material for: Small Mass but Strong Information: Diagnostic Ions Provide Crucial Clues to Correctly Identify Histone Lysine Modifications
Source: Proteomes. 2021 Apr 23;9(2):18. doi: 10.3390/proteomes9020018 (PMC8167651; doi:10.3390/proteomes9020018)
Supplement: Supplementary file 1 [file proteomes-09-00018-s001.zip › proteomes-1166307-supplementary.pptx]

## Slide 1
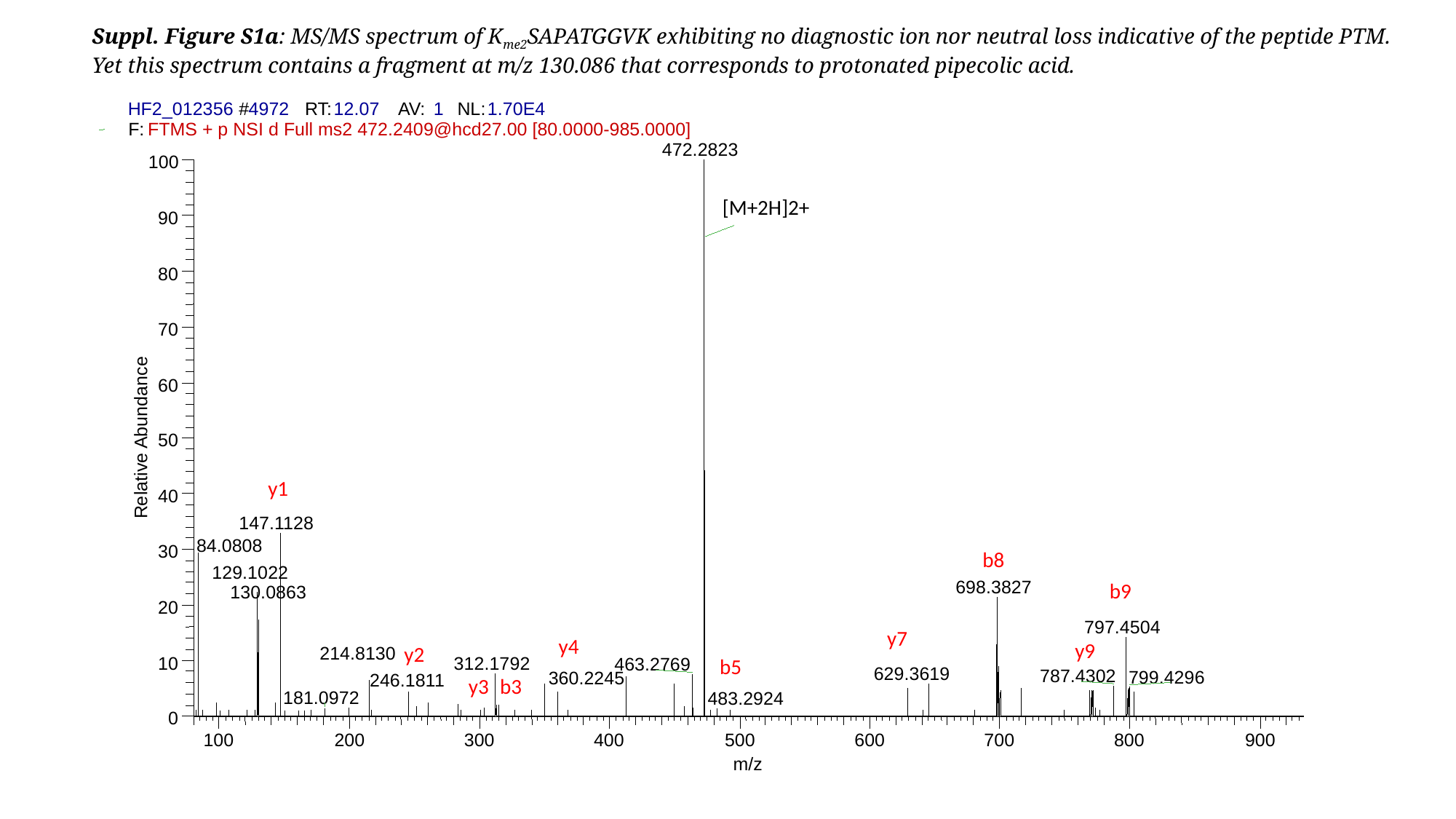

Suppl. Figure S1a: MS/MS spectrum of Kme2SAPATGGVK exhibiting no diagnostic ion nor neutral loss indicative of the peptide PTM.
Yet this spectrum contains a fragment at m/z 130.086 that corresponds to protonated pipecolic acid.
HF2_012356
#
4972
RT:
12.07
AV:
1
NL:
1.70E4
F:
FTMS + p NSI d Full ms2 472.2409@hcd27.00 [80.0000-985.0000]
100
200
300
400
500
600
700
800
900
m/z
472.2823
100
90
80
70
60
Relative Abundance
50
40
147.1128
30
698.3827
20
797.4504
214.8130
10
312.1792
463.2769
629.3619
787.4302
799.4296
181.0972
483.2924
0
[M+2H]2+
y1
84.0808
b8
129.1022
b9
130.0863
y7
y4
y9
y2
b5
360.2245
b3
y3
246.1811

## Slide 2
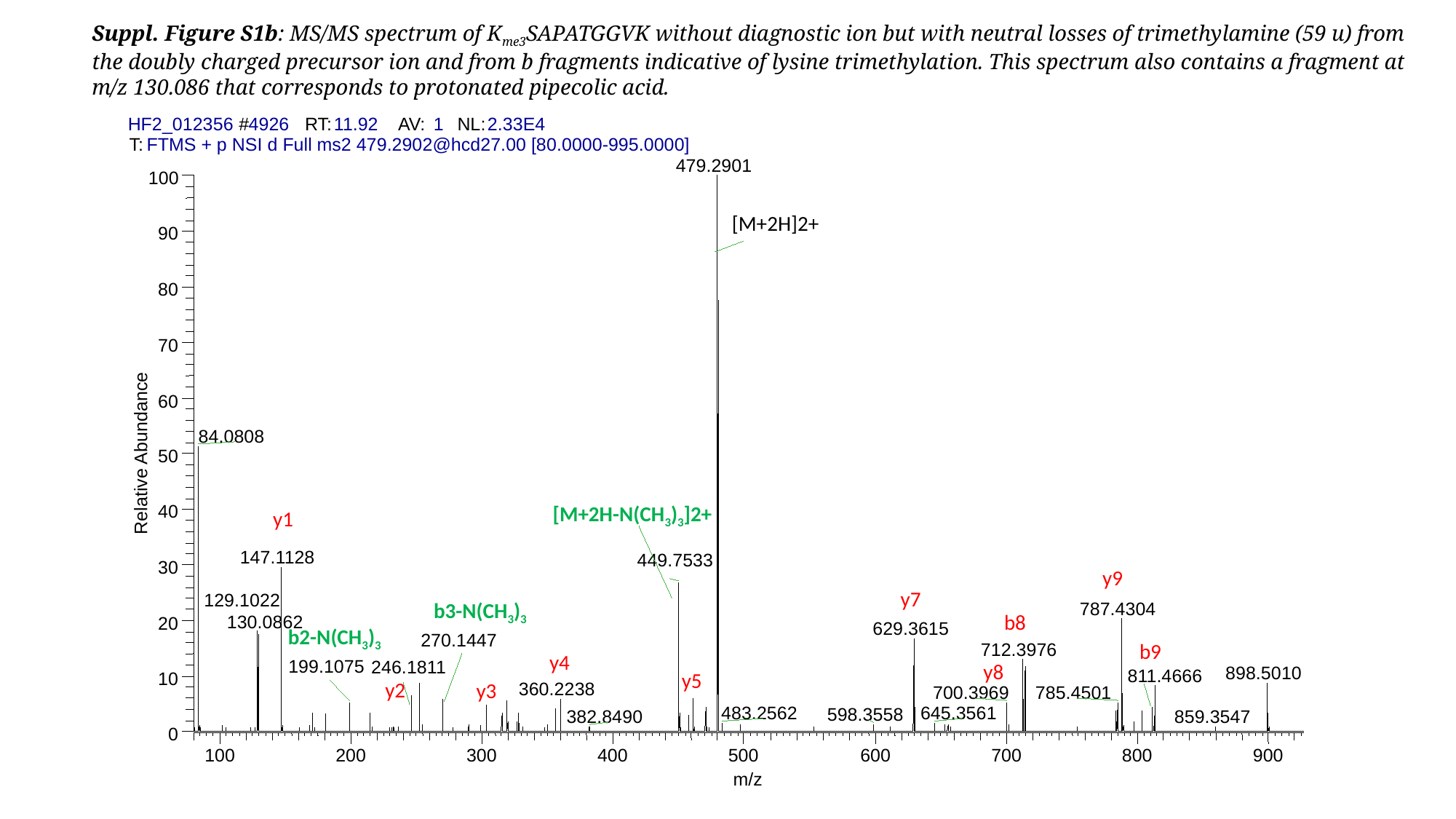

Suppl. Figure S1b: MS/MS spectrum of Kme3SAPATGGVK without diagnostic ion but with neutral losses of trimethylamine (59 u) from the doubly charged precursor ion and from b fragments indicative of lysine trimethylation. This spectrum also contains a fragment at m/z 130.086 that corresponds to protonated pipecolic acid.
HF2_012356
#
4926
RT:
11.92
AV:
1
NL:
2.33E4
T:
FTMS + p NSI d Full ms2 479.2902@hcd27.00 [80.0000-995.0000]
100
200
300
400
500
600
700
800
900
m/z
479.2901
100
90
80
70
60
84.0808
Relative Abundance
50
40
147.1128
449.7533
30
787.4304
20
629.3615
712.3976
199.1075
246.1811
898.5010
811.4666
10
360.2238
700.3969
785.4501
483.2562
645.3561
598.3558
382.8490
859.3547
0
[M+2H]2+
[M+2H-N(CH3)3]2+
y1
y9
y7
129.1022
b3-N(CH3)3
b8
130.0862
b2-N(CH3)3
270.1447
b9
y4
y8
y5
y2
y3

## Slide 3
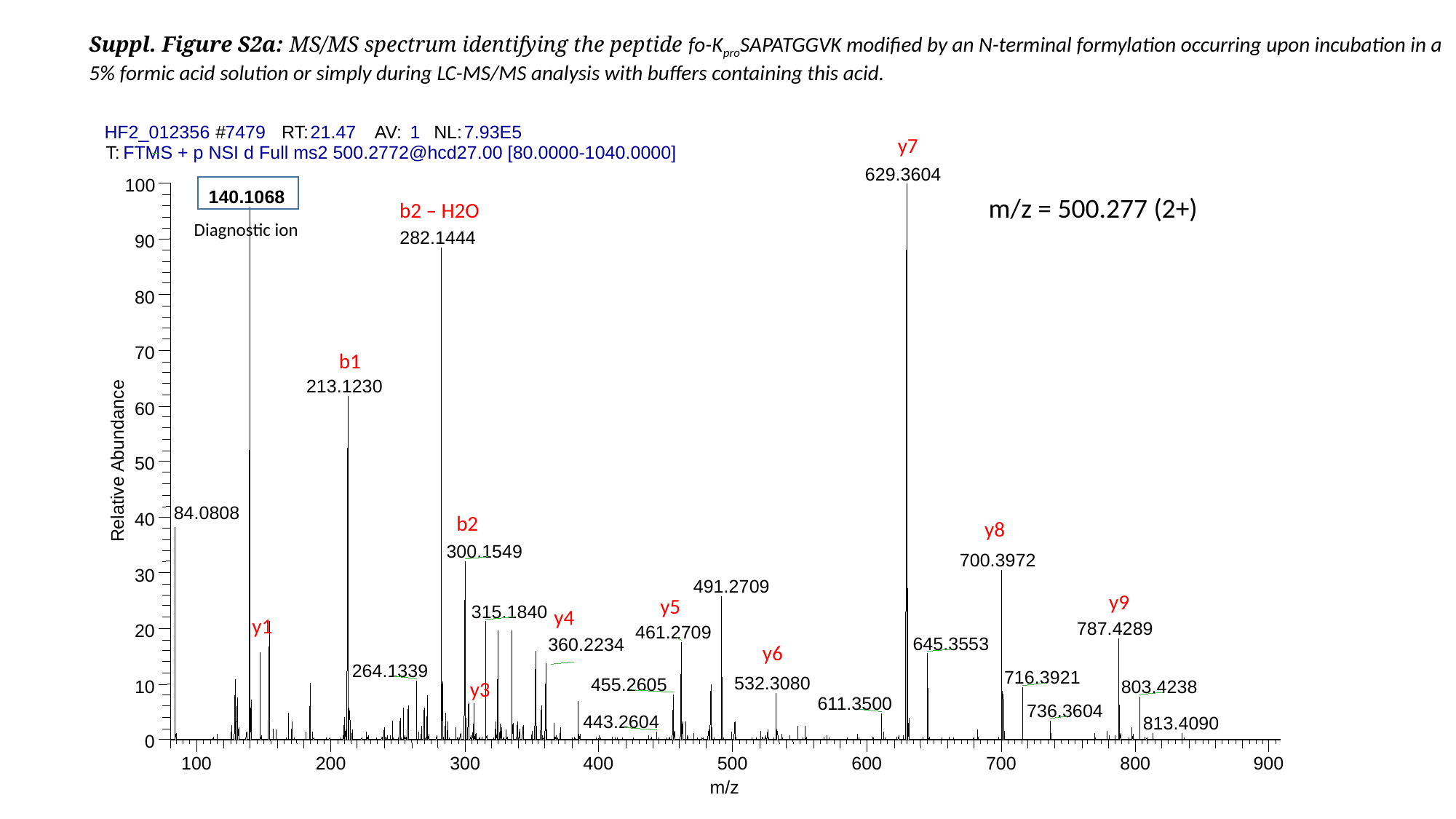

Suppl. Figure S2a: MS/MS spectrum identifying the peptide fo-KproSAPATGGVK modified by an N-terminal formylation occurring upon incubation in a 5% formic acid solution or simply during LC-MS/MS analysis with buffers containing this acid.
HF2_012356
#
7479
RT:
21.47
AV:
1
NL:
7.93E5
T:
FTMS + p NSI d Full ms2 500.2772@hcd27.00 [80.0000-1040.0000]
100
200
300
400
500
600
700
800
900
m/z
629.3604
100
140.1068
282.1444
90
80
70
213.1230
60
Relative Abundance
50
40
300.1549
700.3972
30
491.2709
315.1840
787.4289
20
461.2709
645.3553
360.2234
264.1339
716.3921
532.3080
455.2605
10
803.4238
611.3500
736.3604
443.2604
813.4090
0
y7
m/z = 500.277 (2+)
b2 – H2O
Diagnostic ion
b1
84.0808
b2
y8
y9
y5
y4
y1
y6
y3

## Slide 4
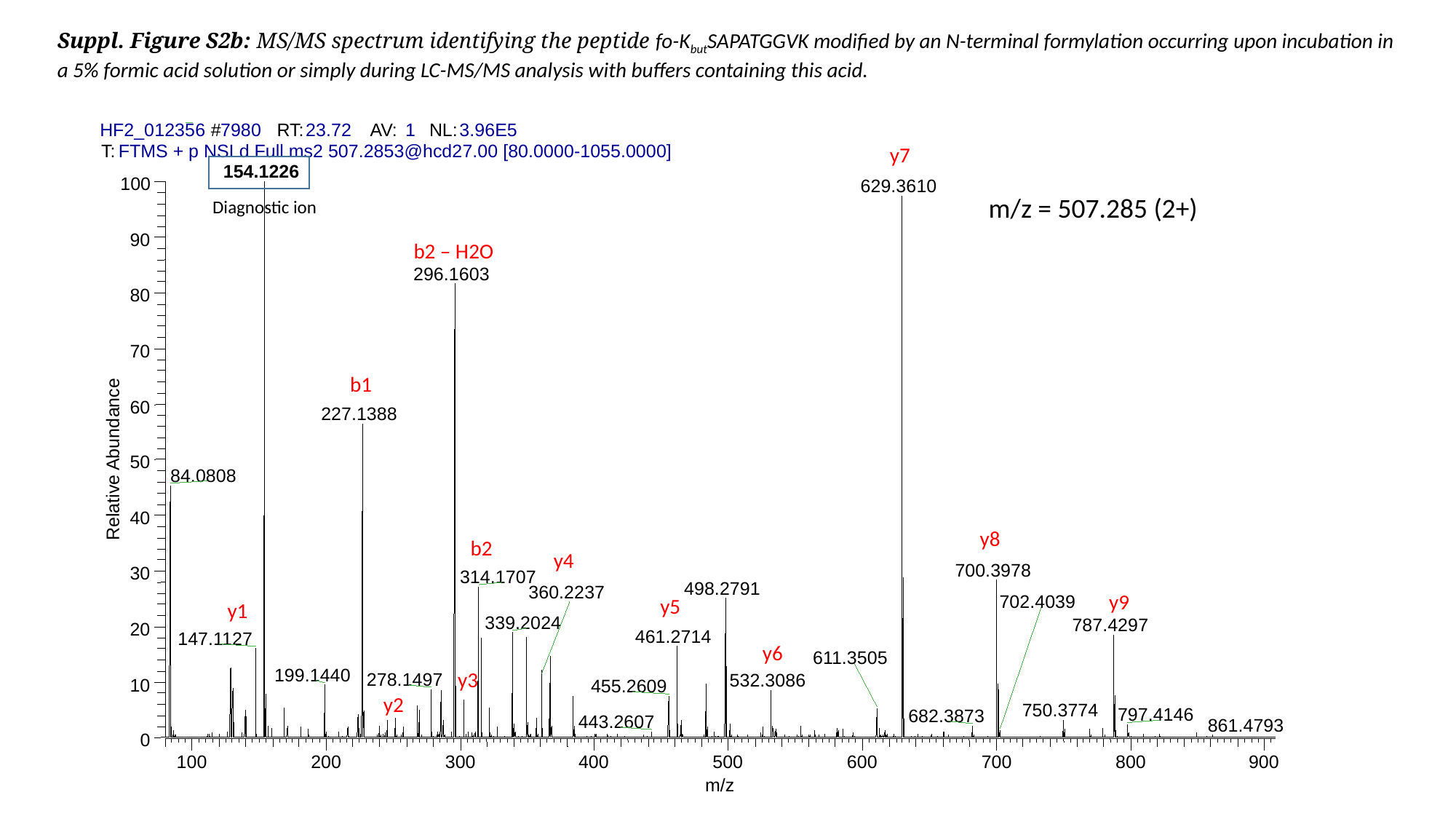

Suppl. Figure S2b: MS/MS spectrum identifying the peptide fo-KbutSAPATGGVK modified by an N-terminal formylation occurring upon incubation in a 5% formic acid solution or simply during LC-MS/MS analysis with buffers containing this acid.
HF2_012356
#
7980
RT:
23.72
AV:
1
NL:
3.96E5
T:
FTMS + p NSI d Full ms2 507.2853@hcd27.00 [80.0000-1055.0000]
100
200
300
400
500
600
700
800
900
m/z
154.1226
100
629.3610
90
296.1603
80
70
60
227.1388
Relative Abundance
50
84.0808
40
700.3978
30
314.1707
498.2791
360.2237
702.4039
339.2024
787.4297
20
461.2714
147.1127
611.3505
199.1440
278.1497
532.3086
10
455.2609
750.3774
797.4146
682.3873
443.2607
861.4793
0
y7
m/z = 507.285 (2+)
Diagnostic ion
b2 – H2O
b1
y8
b2
y4
y9
y5
y1
y6
y3
y2

## Slide 5
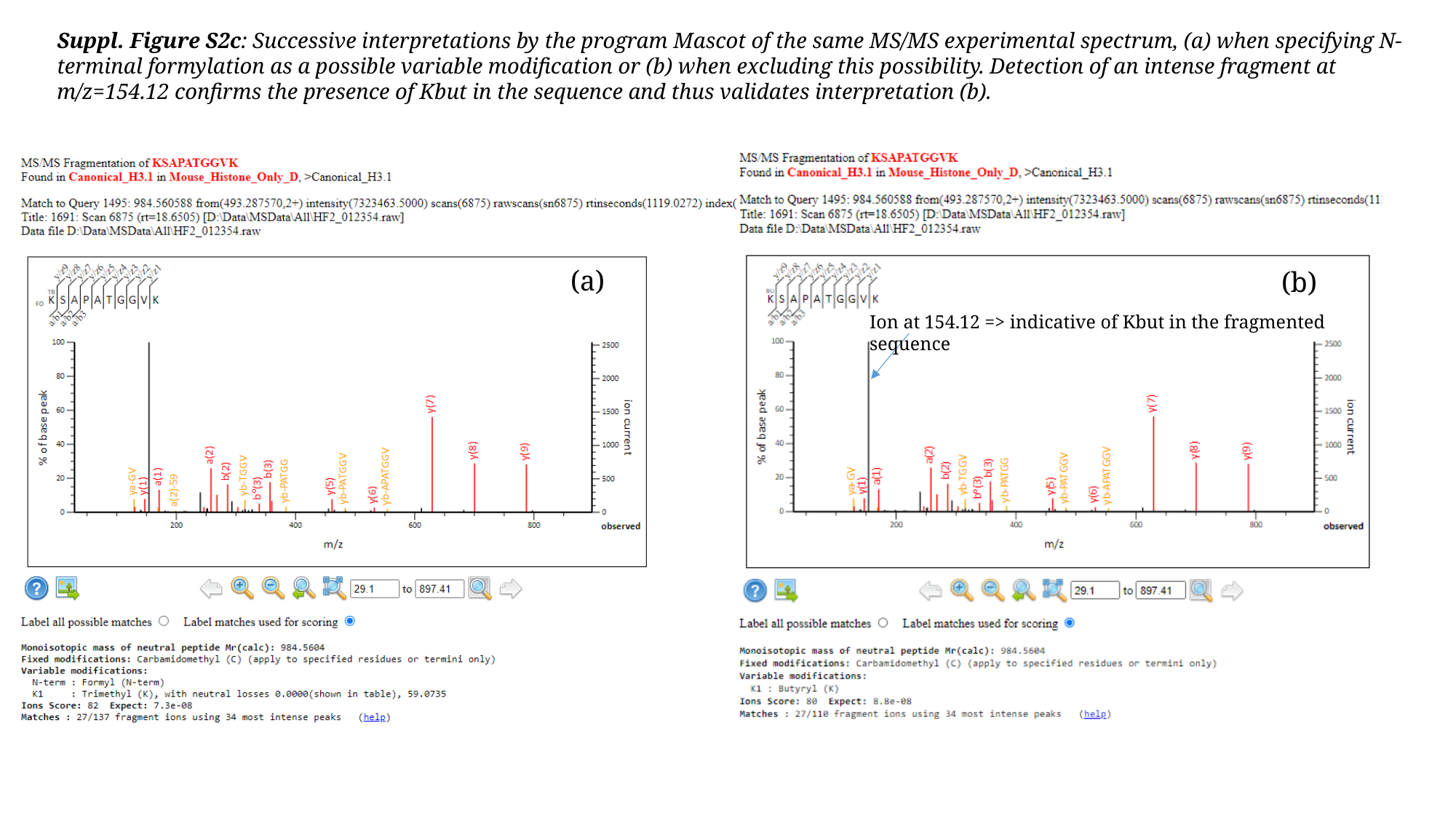

Suppl. Figure S2c: Successive interpretations by the program Mascot of the same MS/MS experimental spectrum, (a) when specifying N-terminal formylation as a possible variable modification or (b) when excluding this possibility. Detection of an intense fragment at m/z=154.12 confirms the presence of Kbut in the sequence and thus validates interpretation (b).
(a)
(b)
Ion at 154.12 => indicative of Kbut in the fragmented sequence

## Slide 6
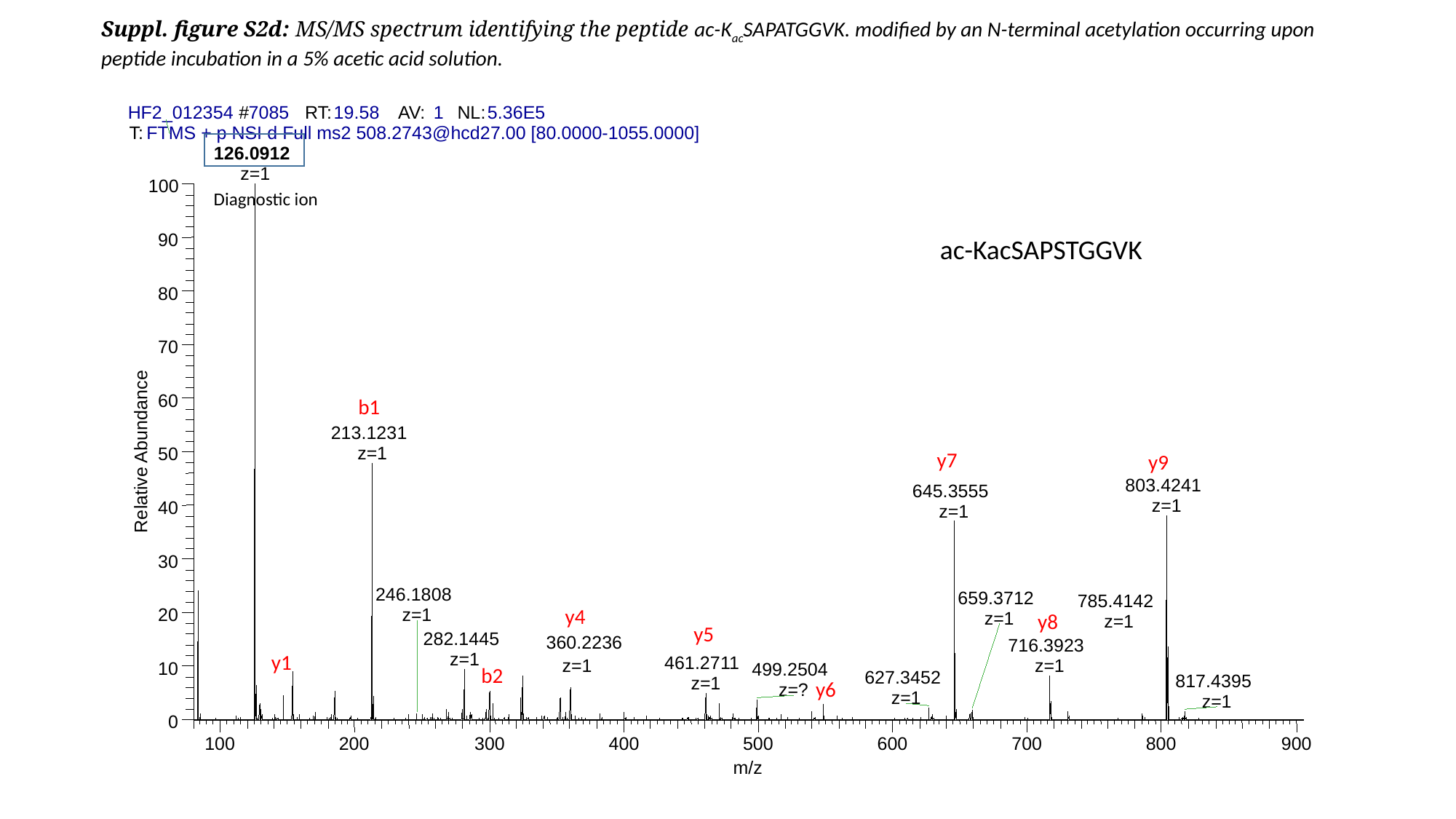

Suppl. figure S2d: MS/MS spectrum identifying the peptide ac-KacSAPATGGVK. modified by an N-terminal acetylation occurring upon peptide incubation in a 5% acetic acid solution.
HF2_012354
#
7085
RT:
19.58
AV:
1
NL:
5.36E5
T:
FTMS + p NSI d Full ms2 508.2743@hcd27.00 [80.0000-1055.0000]
100
200
300
400
500
600
700
800
900
m/z
126.0912
z=1
100
90
80
70
60
213.1231
Relative Abundance
z=1
50
803.4241
645.3555
z=1
40
z=1
30
246.1808
659.3712
785.4142
20
z=1
z=1
z=1
282.1445
360.2236
716.3923
z=1
461.2711
z=1
z=1
10
499.2504
627.3452
817.4395
z=1
z=?
z=1
z=1
0
Diagnostic ion
ac-KacSAPSTGGVK
b1
y7
y9
y4
y8
y5
y1
b2
y6

## Slide 7
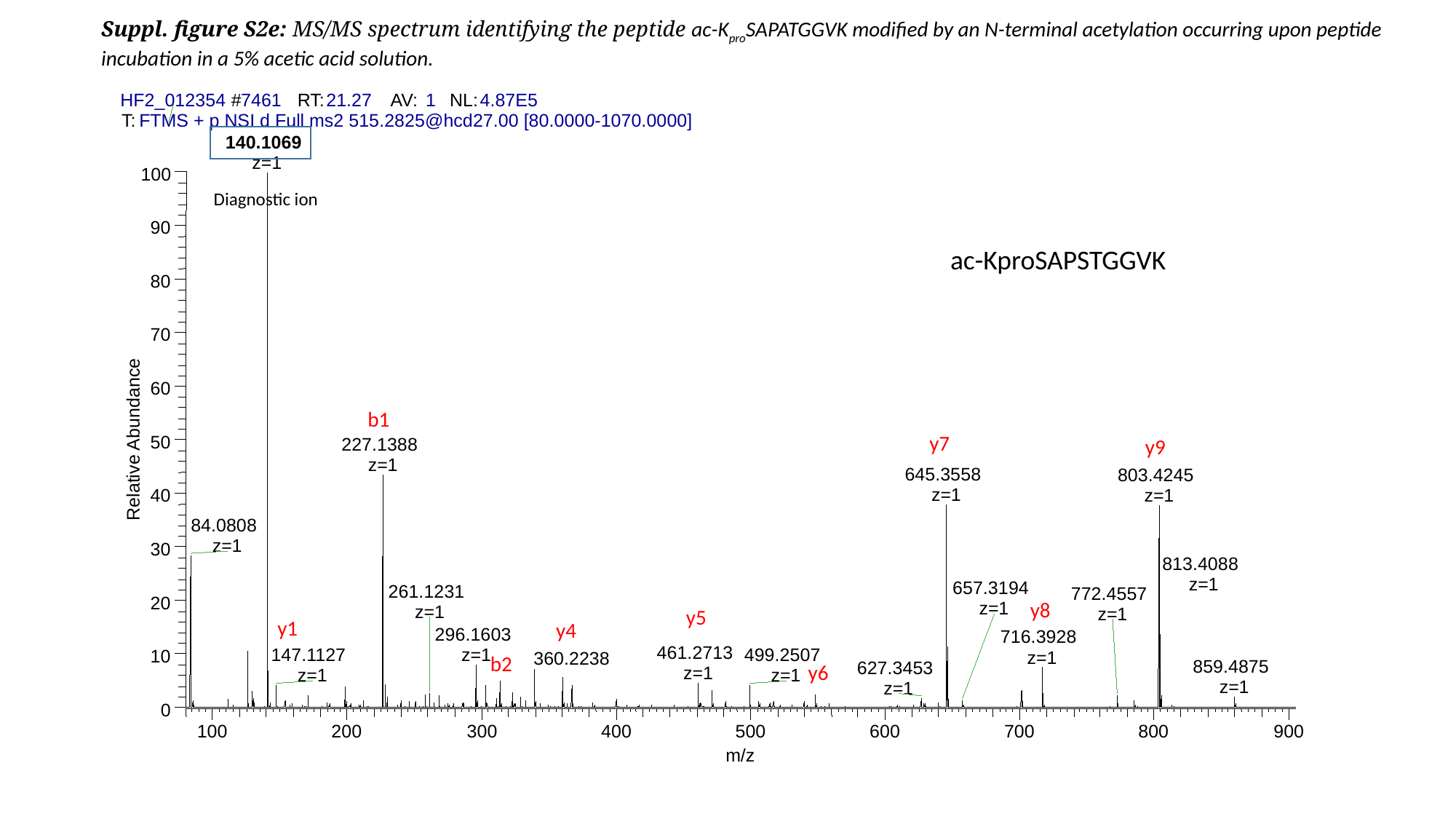

Suppl. figure S2e: MS/MS spectrum identifying the peptide ac-KproSAPATGGVK modified by an N-terminal acetylation occurring upon peptide incubation in a 5% acetic acid solution.
HF2_012354
#
7461
RT:
21.27
AV:
1
NL:
4.87E5
T:
FTMS + p NSI d Full ms2 515.2825@hcd27.00 [80.0000-1070.0000]
100
200
300
400
500
600
700
800
900
m/z
140.1069
z=1
100
90
80
70
60
Relative Abundance
50
227.1388
z=1
645.3558
803.4245
z=1
40
z=1
84.0808
z=1
30
813.4088
z=1
657.3194
261.1231
772.4557
20
z=1
z=1
z=1
296.1603
716.3928
461.2713
147.1127
z=1
499.2507
10
z=1
360.2238
859.4875
627.3453
z=1
z=1
z=1
z=1
z=1
0
Diagnostic ion
ac-KproSAPSTGGVK
b1
y7
y9
y8
y5
y1
y4
b2
y6

## Slide 8
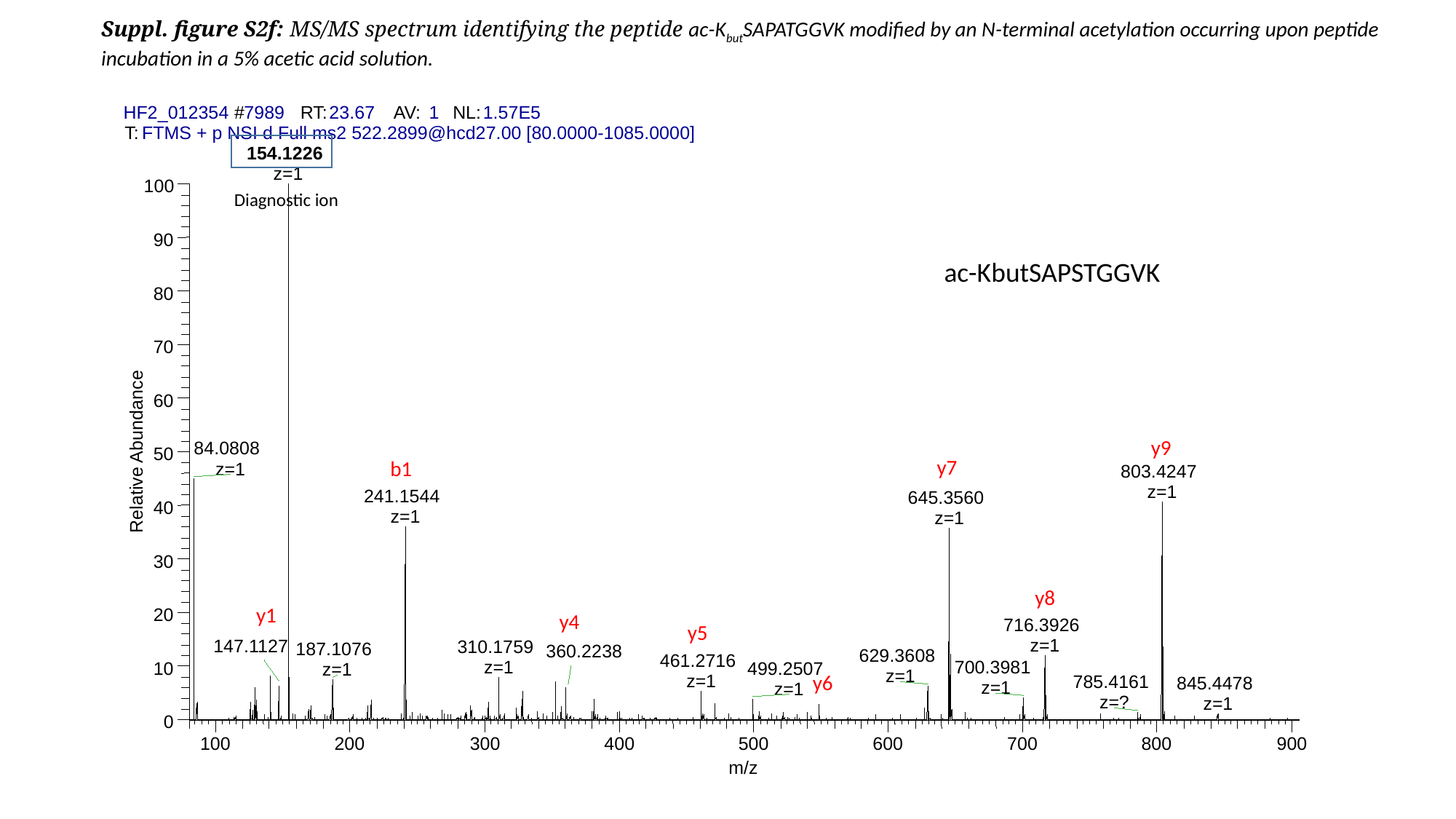

Suppl. figure S2f: MS/MS spectrum identifying the peptide ac-KbutSAPATGGVK modified by an N-terminal acetylation occurring upon peptide incubation in a 5% acetic acid solution.
HF2_012354
#
7989
RT:
23.67
AV:
1
NL:
1.57E5
T:
FTMS + p NSI d Full ms2 522.2899@hcd27.00 [80.0000-1085.0000]
100
200
300
400
500
600
700
800
900
m/z
154.1226
z=1
100
90
80
70
60
84.0808
Relative Abundance
50
z=1
803.4247
z=1
241.1544
645.3560
40
z=1
z=1
30
20
716.3926
z=1
147.1127
310.1759
187.1076
360.2238
629.3608
461.2716
z=1
700.3981
10
499.2507
z=1
z=1
z=1
785.4161
845.4478
z=1
z=1
z=?
z=1
0
Diagnostic ion
ac-KbutSAPSTGGVK
y9
y7
b1
y8
y1
y4
y5
y6

## Slide 9
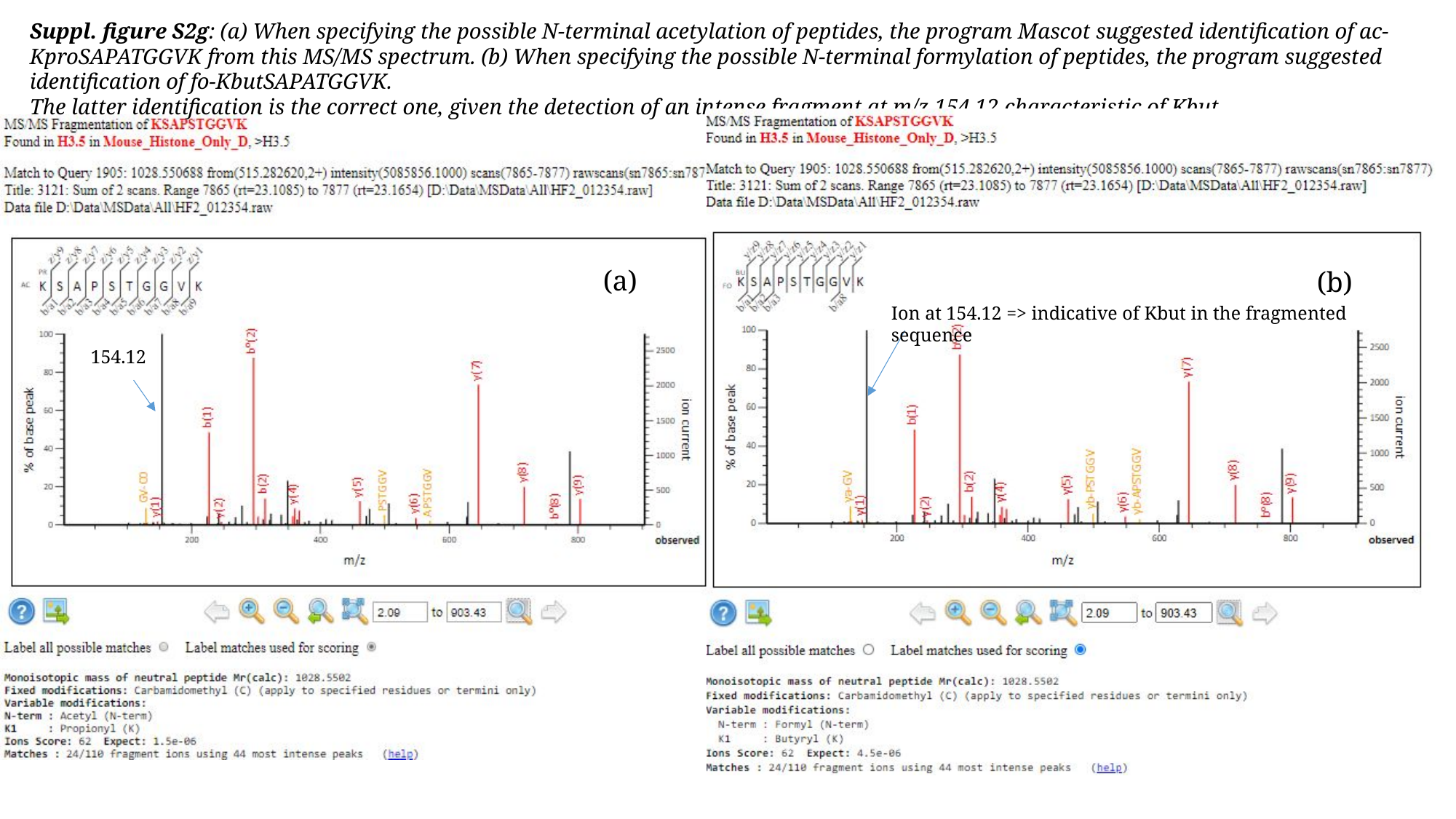

Suppl. figure S2g: (a) When specifying the possible N-terminal acetylation of peptides, the program Mascot suggested identification of ac-KproSAPATGGVK from this MS/MS spectrum. (b) When specifying the possible N-terminal formylation of peptides, the program suggested identification of fo-KbutSAPATGGVK.
The latter identification is the correct one, given the detection of an intense fragment at m/z 154.12 characteristic of Kbut.
(a)
(b)
Ion at 154.12 => indicative of Kbut in the fragmented sequence
154.12

## Slide 10
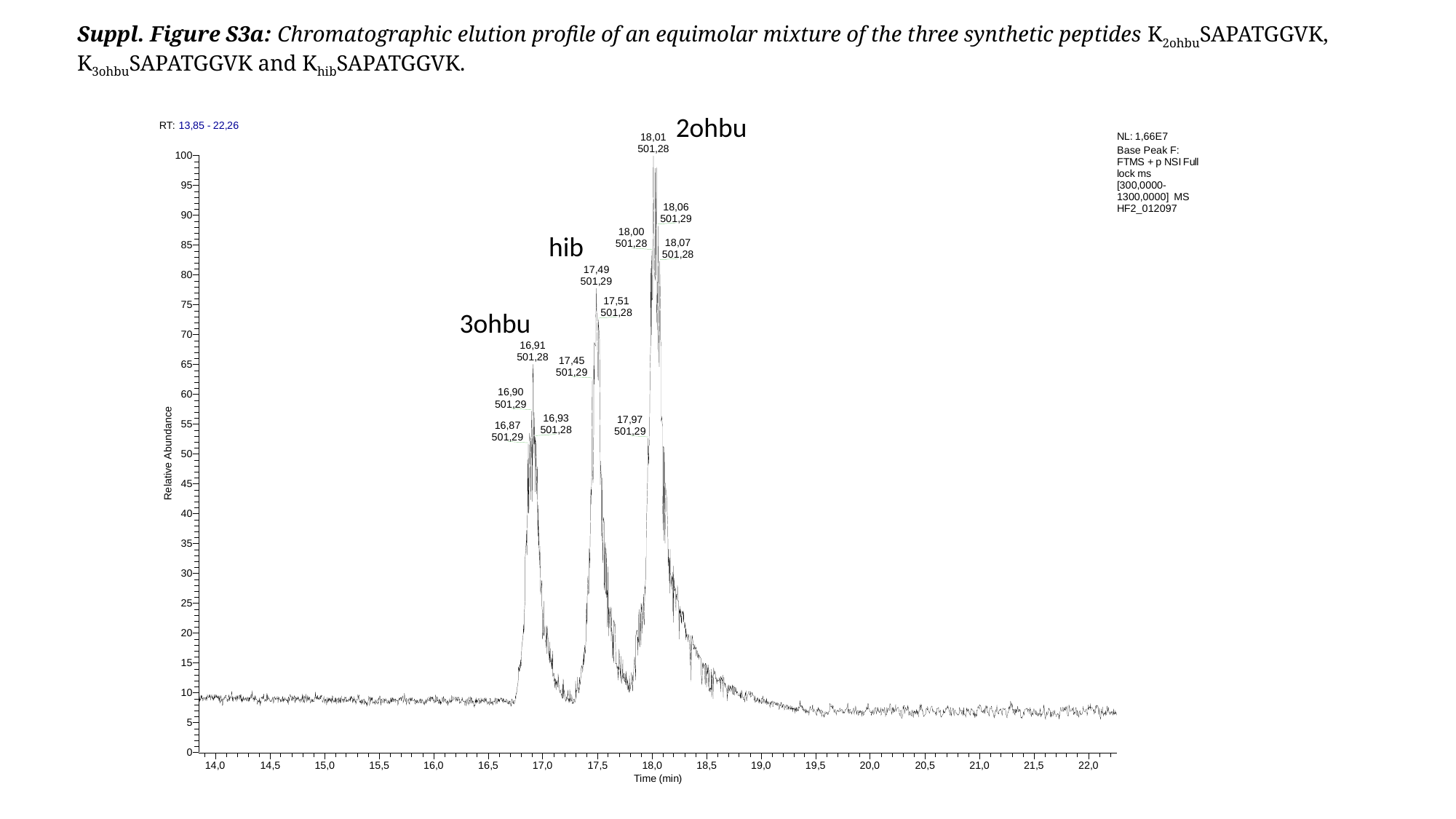

Suppl. Figure S3a: Chromatographic elution profile of an equimolar mixture of the three synthetic peptides K2ohbuSAPATGGVK, K3ohbuSAPATGGVK and KhibSAPATGGVK.
2ohbu
hib
3ohbu

## Slide 11
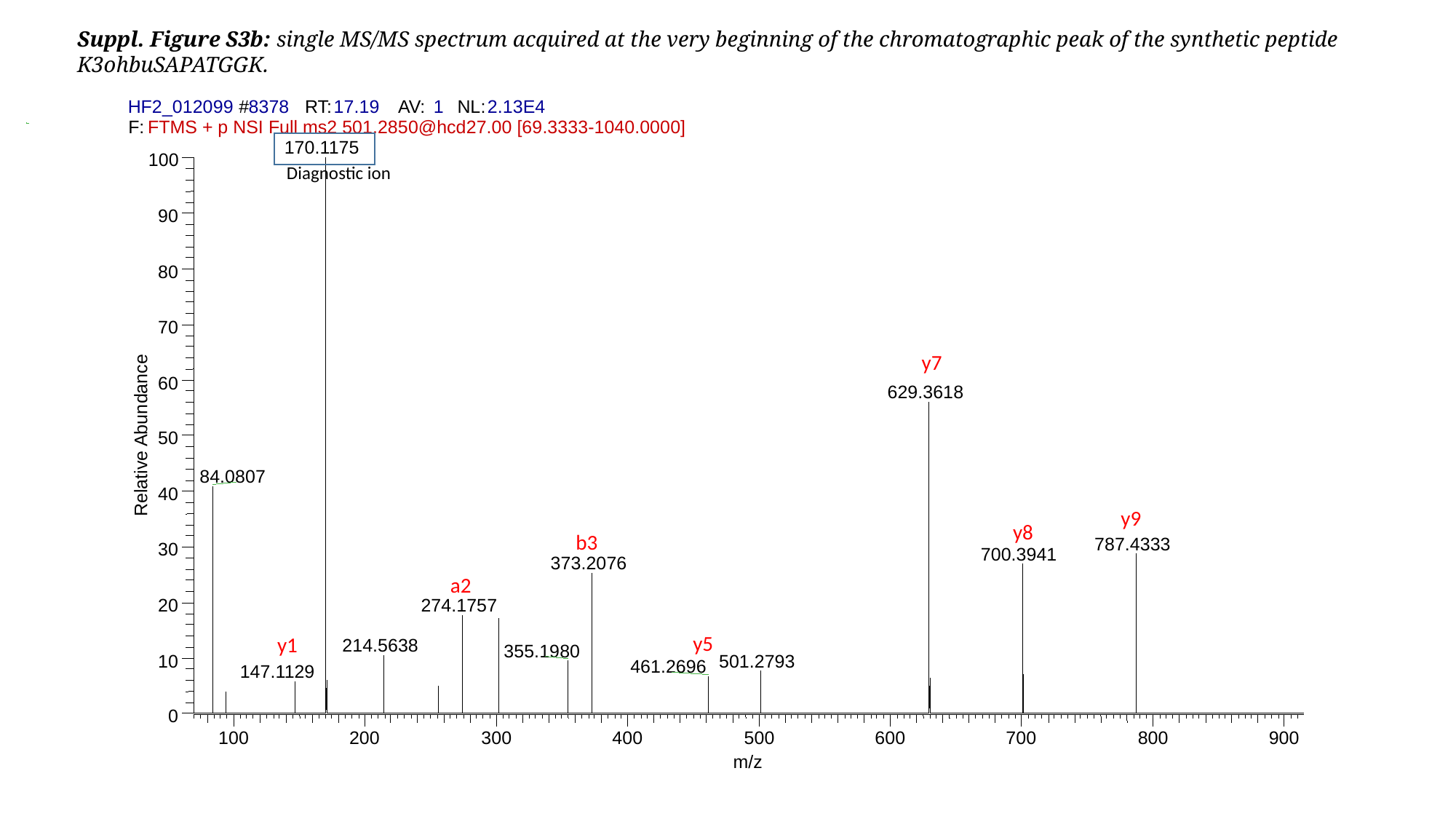

Suppl. Figure S3b: single MS/MS spectrum acquired at the very beginning of the chromatographic peak of the synthetic peptide K3ohbuSAPATGGK.
HF2_012099
#
8378
RT:
17.19
AV:
1
NL:
2.13E4
F:
FTMS + p NSI Full ms2 501.2850@hcd27.00 [69.3333-1040.0000]
100
200
300
400
500
600
700
800
900
m/z
170.1175
100
90
80
70
60
629.3618
Relative Abundance
50
84.0807
40
787.4333
30
700.3941
373.2076
20
274.1757
214.5638
355.1980
10
501.2793
461.2696
147.1129
0
Diagnostic ion
y7
y9
y8
b3
a2
y5
y1

## Slide 12
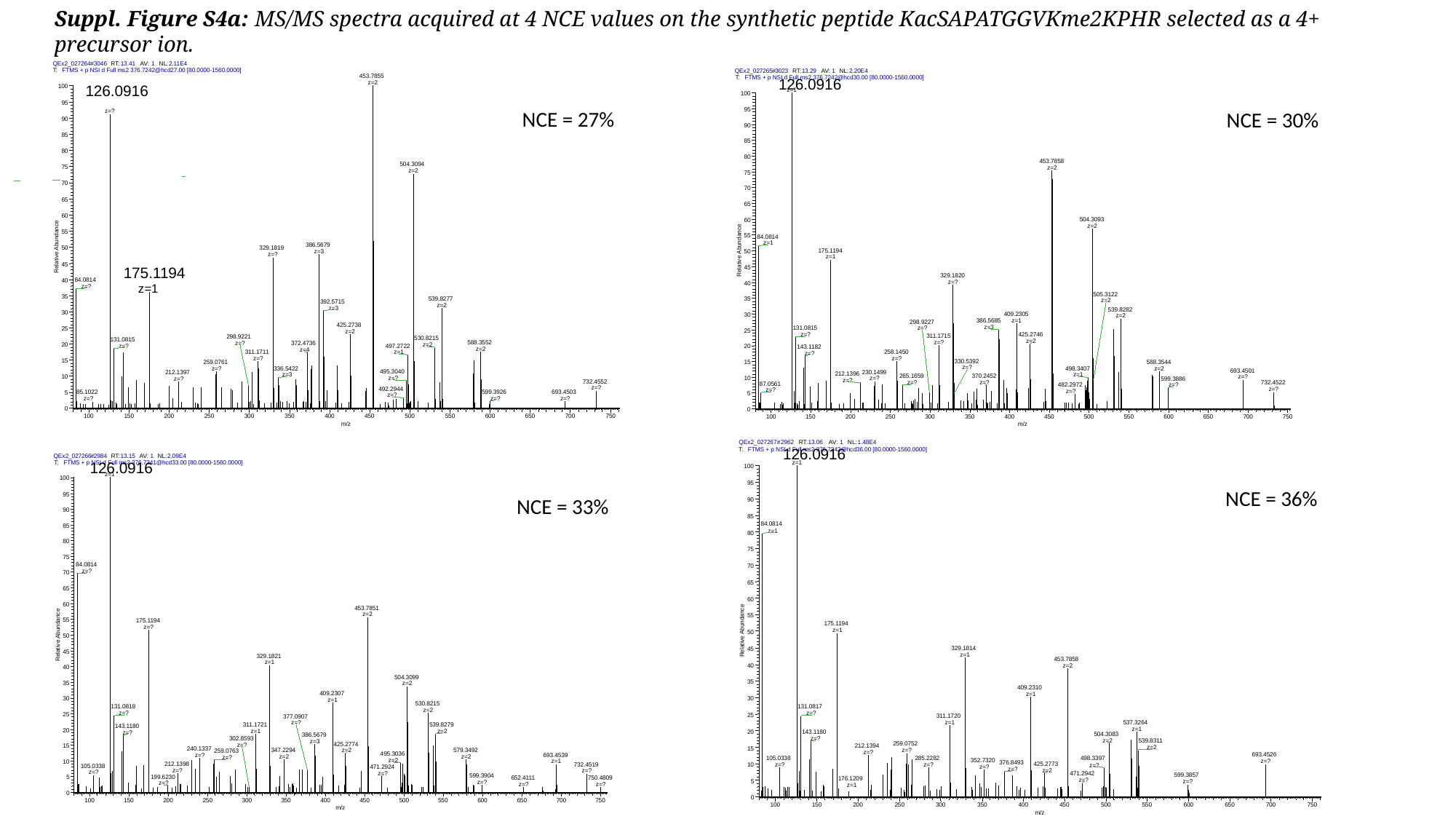

Suppl. Figure S4a: MS/MS spectra acquired at 4 NCE values on the synthetic peptide KacSAPATGGVKme2KPHR selected as a 4+ precursor ion.
QEx2_027264
#
3046
RT:
13.41
AV:
1
NL:
2.11E4
T:
FTMS + p NSI d Full ms2 376.7242@hcd27.00 [80.0000-1560.0000]
453.7855
z=2
126.0916
100
100
150
200
250
300
350
400
450
500
550
600
650
700
750
m/z
95
z=?
90
85
80
504.3094
75
z=2
70
65
60
55
386.5679
Relative Abundance
50
329.1819
z=3
z=?
45
175.1194
84.0814
40
z=1
z=?
35
539.8277
392.5715
z=2
z=3
30
425.2738
25
z=2
298.9221
530.8215
131.0815
588.3552
z=?
372.4736
20
z=2
z=?
497.2722
z=2
z=4
311.1711
z=1
z=?
15
259.0761
336.5422
z=?
495.3040
212.1397
z=3
10
z=?
z=?
732.4552
z=?
492.2944
85.1022
599.3926
693.4503
5
z=?
z=?
z=?
z=?
0
QEx2_027265
#
3023
RT:
13.29
AV:
1
NL:
2.20E4
T:
FTMS + p NSI d Full ms2 376.7242@hcd30.00 [80.0000-1560.0000]
126.0916
z=1
100
100
150
200
250
300
350
400
450
500
550
600
650
700
750
m/z
95
90
85
80
453.7858
z=2
75
70
65
504.3093
60
z=2
55
84.0814
z=1
Relative Abundance
175.1194
50
z=1
45
329.1820
z=?
40
505.3122
35
z=2
539.8282
409.2305
30
z=2
z=1
386.5685
298.9227
z=3
131.0815
z=?
25
z=?
425.2746
311.1715
z=2
z=?
20
143.1182
258.1450
z=?
z=?
330.5392
15
588.3544
z=?
498.3407
z=2
693.4501
230.1499
212.1396
z=1
370.2452
265.1659
z=?
10
z=?
599.3886
z=?
z=?
z=?
732.4522
87.0561
482.2972
z=?
z=?
z=?
z=?
5
0
NCE = 27%
NCE = 30%
QEx2_027267
#
2962
RT:
13.06
AV:
1
NL:
1.48E4
T:
FTMS + p NSI d Full ms2 376.7242@hcd36.00 [80.0000-1560.0000]
126.0916
z=1
100
100
150
200
250
300
350
400
450
500
550
600
650
700
750
m/z
95
90
85
84.0814
z=1
80
75
70
65
60
55
175.1194
Relative Abundance
z=1
50
329.1814
45
z=1
453.7858
40
z=2
35
409.2310
z=1
30
131.0817
z=?
25
311.1720
537.3264
z=1
z=1
20
143.1180
504.3083
z=?
z=2
539.8311
259.0752
212.1394
z=2
15
z=?
z=?
693.4526
105.0338
285.2282
498.3397
352.7320
z=?
376.8493
425.2773
10
z=?
z=?
z=?
z=?
z=?
z=2
471.2942
599.3857
176.1209
z=?
5
z=?
z=1
0
QEx2_027266
#
2984
RT:
13.15
AV:
1
NL:
2.09E4
T:
FTMS + p NSI d Full ms2 376.7241@hcd33.00 [80.0000-1560.0000]
126.0916
z=1
100
100
150
200
250
300
350
400
450
500
550
600
650
700
750
m/z
95
90
85
80
75
84.0814
z=?
70
65
60
453.7851
z=2
55
175.1194
z=?
Relative Abundance
50
45
329.1821
z=1
40
504.3099
35
z=2
409.2307
30
z=1
530.8215
131.0818
z=2
z=?
25
377.0907
z=?
311.1721
539.8279
143.1180
20
z=1
z=2
z=?
386.5679
302.8593
z=3
425.2774
z=?
15
240.1337
579.3492
347.2294
z=2
259.0763
495.3036
z=?
693.4539
z=2
z=2
z=?
z=2
z=1
10
212.1398
732.4519
105.0338
471.2924
z=?
z=?
z=?
z=?
599.3904
199.6230
5
652.4111
750.4809
z=?
z=?
z=?
z=?
0
NCE = 36%
NCE = 33%

## Slide 13
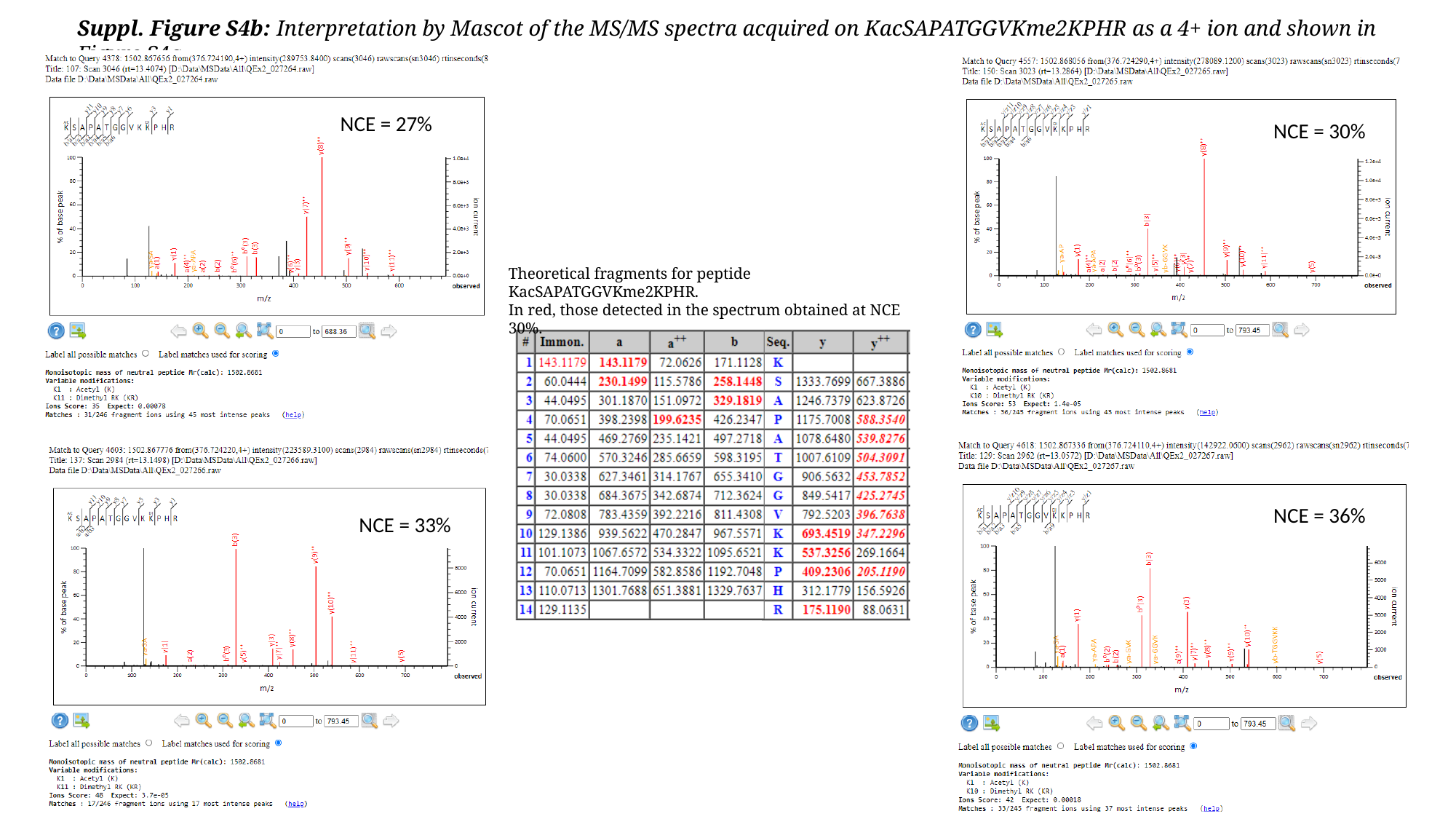

Suppl. Figure S4b: Interpretation by Mascot of the MS/MS spectra acquired on KacSAPATGGVKme2KPHR as a 4+ ion and shown in Figure S4a.
NCE = 27%
NCE = 30%
Theoretical fragments for peptide KacSAPATGGVKme2KPHR.
In red, those detected in the spectrum obtained at NCE 30%.
NCE = 36%
NCE = 33%

## Slide 14
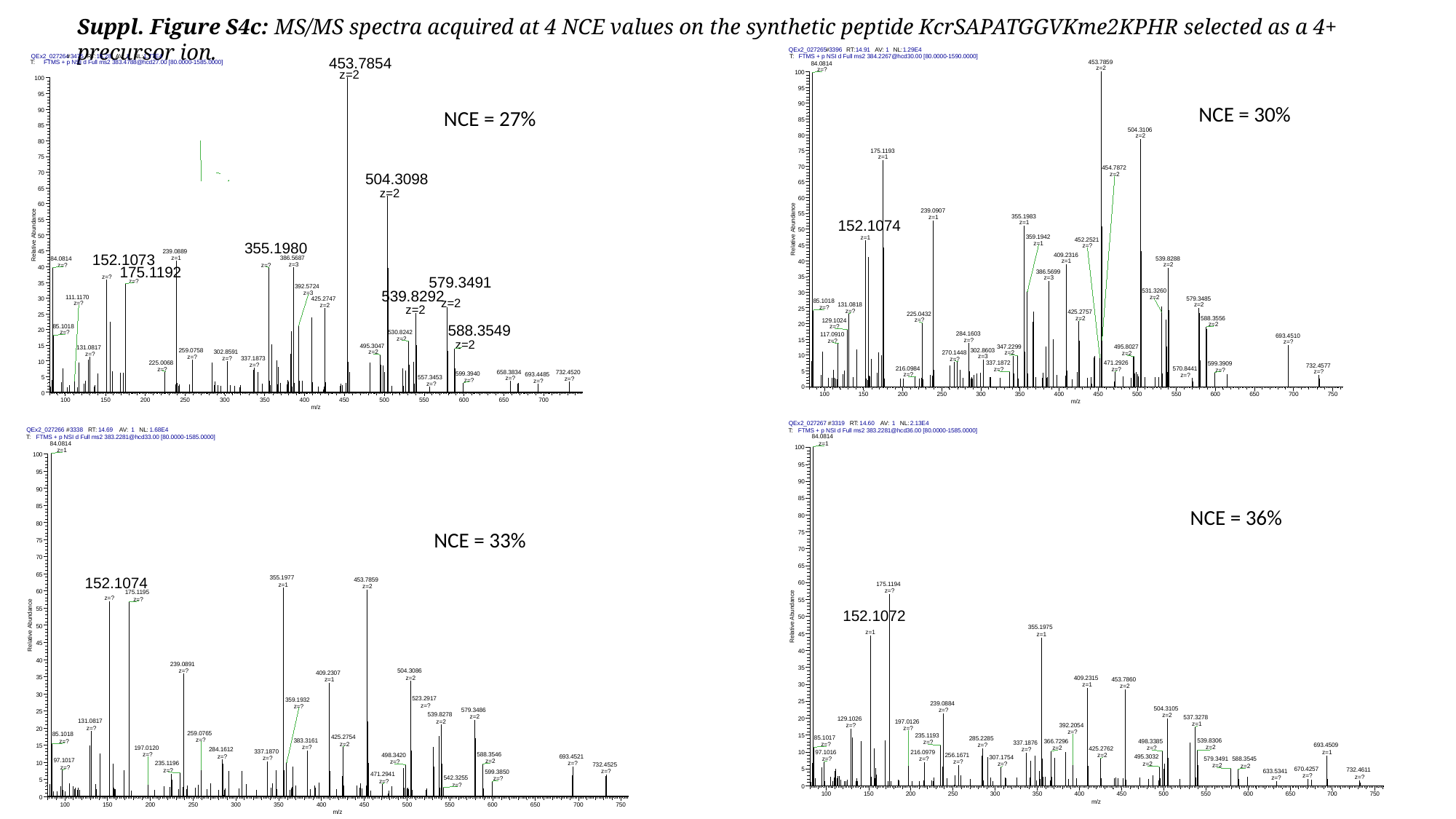

Suppl. Figure S4c: MS/MS spectra acquired at 4 NCE values on the synthetic peptide KcrSAPATGGVKme2KPHR selected as a 4+ precursor ion.
QEx2_027265
#
3396
RT:
14.91
AV:
1
NL:
1.29E4
T:
FTMS + p NSI d Full ms2 384.2267@hcd30.00 [80.0000-1590.0000]
QEx2_027264
#
3475
RT:
15.28
AV:
1
NL:
1.77E4
T:
FTMS + p NSI d Full ms2 383.4788@hcd27.00 [80.0000-1585.0000]
453.7854
z=2
100
100
150
200
250
300
350
400
450
500
550
600
650
700
m/z
95
90
85
80
75
70
504.3098
65
z=2
60
55
Relative Abundance
50
355.1980
45
239.0889
152.1073
z=1
386.5687
84.0814
z=3
z=?
z=?
175.1192
40
579.3491
z=?
z=?
35
392.5724
539.8292
z=3
111.1170
30
425.2747
z=2
z=?
z=2
z=2
25
588.3549
85.1018
20
530.8242
z=?
z=2
z=2
15
495.3047
131.0817
259.0758
302.8591
z=2
z=?
z=?
z=?
337.1873
10
225.0068
z=?
z=?
658.3834
732.4520
599.3940
693.4485
5
557.3453
z=?
z=?
z=?
z=?
z=?
0
453.7859
84.0814
z=2
z=?
100
100
150
200
250
300
350
400
450
500
550
600
650
700
750
m/z
95
NCE = 30%
NCE = 27%
90
85
504.3106
80
z=2
QEx2_027266
#
3338
RT:
14.69
AV:
1
NL:
1.68E4
T:
FTMS + p NSI d Full ms2 383.2281@hcd33.00 [80.0000-1585.0000]
84.0814
z=1
100
100
150
200
250
300
350
400
450
500
550
600
650
700
750
m/z
95
90
85
80
75
70
65
152.1074
355.1977
453.7859
z=1
z=2
60
175.1195
z=?
z=?
55
Relative Abundance
50
45
40
239.0891
z=?
504.3086
409.2307
35
z=2
z=1
30
523.2917
359.1932
z=?
z=?
579.3486
25
539.8278
z=2
131.0817
z=2
z=?
20
259.0765
85.1018
425.2754
z=?
383.3161
z=?
z=2
15
z=?
197.0120
284.1612
337.1870
588.3546
z=?
498.3420
z=?
693.4521
z=?
97.1017
z=2
z=?
10
235.1196
z=?
732.4525
z=?
z=?
z=?
599.3850
471.2941
542.3255
z=?
5
z=?
z=?
0
175.1193
75
z=1
70
454.7872
z=2
65
60
239.0907
55
355.1983
z=1
152.1074
z=1
Relative Abundance
50
359.1942
z=1
452.2521
z=1
45
z=?
409.2316
539.8288
z=1
40
z=2
386.5699
35
z=3
531.3260
30
z=2
579.3485
85.1018
131.0818
z=2
z=?
25
z=?
425.2757
225.0432
z=2
588.3556
z=?
129.1024
20
z=2
z=?
284.1603
117.0910
693.4510
15
z=?
z=?
z=?
347.2299
495.8027
302.8603
270.1448
z=2
z=2
10
z=3
z=?
471.2926
337.1872
599.3909
732.4577
216.0984
570.8441
z=?
z=?
z=?
5
z=?
z=?
z=?
0
QEx2_027267
#
3319
RT:
14.60
AV:
1
NL:
2.13E4
T:
FTMS + p NSI d Full ms2 383.2281@hcd36.00 [80.0000-1585.0000]
84.0814
z=1
100
100
150
200
250
300
350
400
450
500
550
600
650
700
750
m/z
95
90
85
80
75
70
65
60
175.1194
z=?
55
152.1072
Relative Abundance
50
355.1975
z=1
45
z=1
40
35
409.2315
453.7860
z=1
30
z=2
25
239.0884
504.3105
z=?
z=2
537.3278
20
129.1026
197.0126
z=1
z=?
392.2054
z=?
z=?
15
235.1193
85.1017
285.2285
539.8306
366.7296
498.3385
z=?
337.1876
z=?
z=?
693.4509
z=2
z=2
z=?
425.2762
z=?
97.1016
z=1
10
216.0979
256.1671
z=2
495.3032
307.1754
z=?
579.3491
z=?
588.3545
z=?
z=2
z=?
z=2
z=2
670.4257
5
732.4611
633.5341
z=?
z=?
z=?
0
NCE = 36%
NCE = 33%

## Slide 15
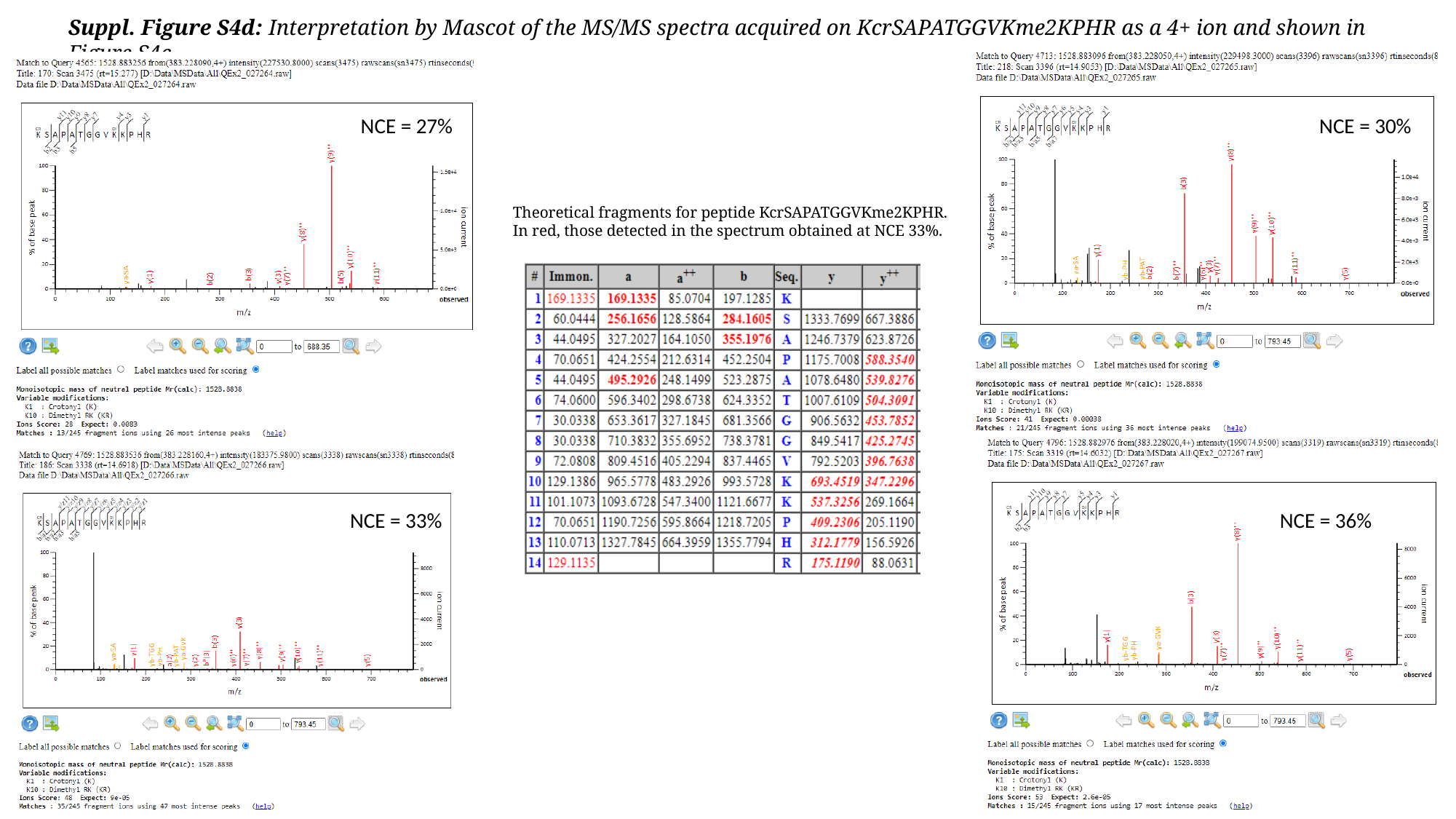

Suppl. Figure S4d: Interpretation by Mascot of the MS/MS spectra acquired on KcrSAPATGGVKme2KPHR as a 4+ ion and shown in Figure S4c.
NCE = 30%
NCE = 27%
Theoretical fragments for peptide KcrSAPATGGVKme2KPHR.
In red, those detected in the spectrum obtained at NCE 33%.
NCE = 36%
NCE = 33%

## Slide 16
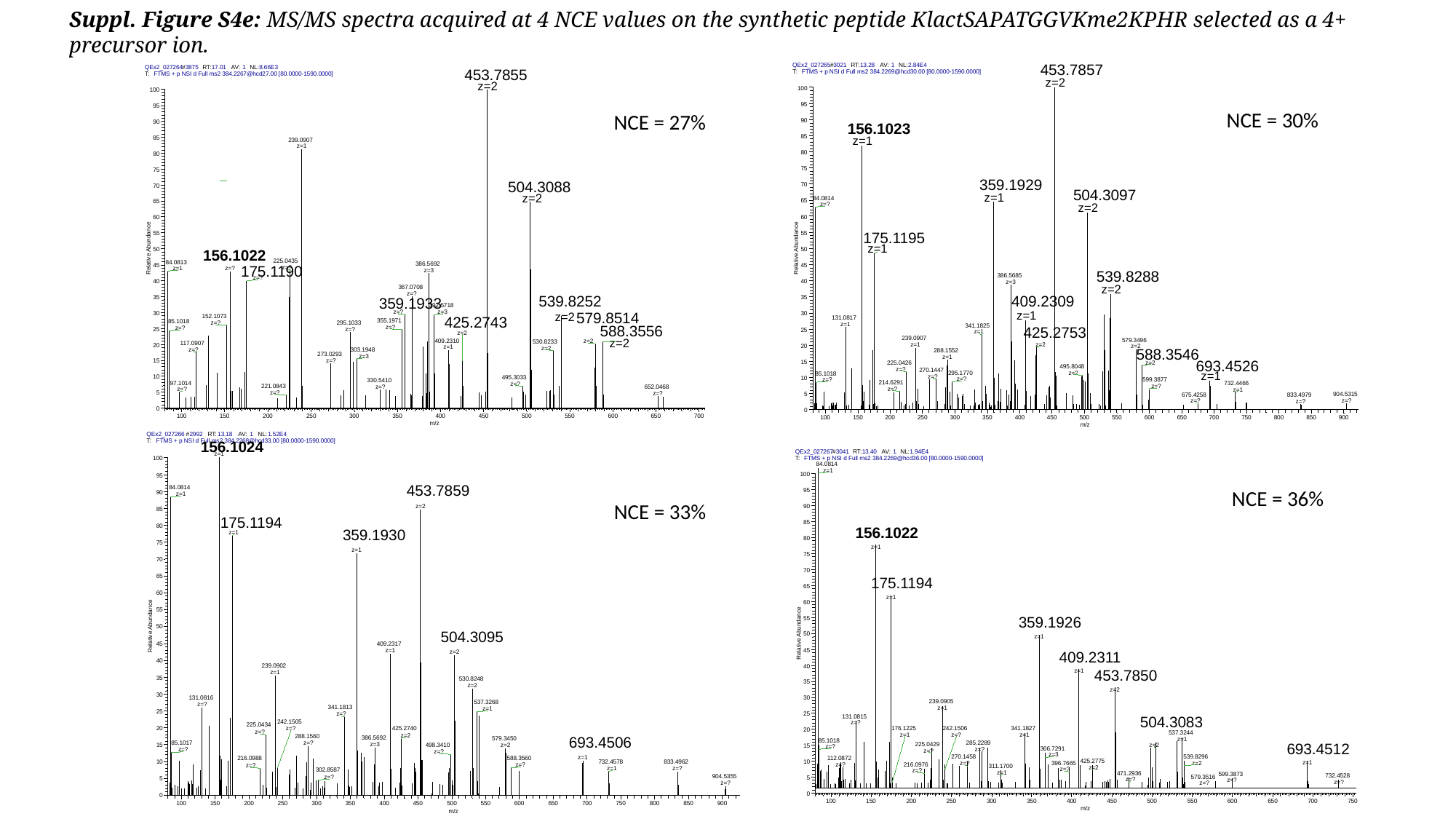

Suppl. Figure S4e: MS/MS spectra acquired at 4 NCE values on the synthetic peptide KlactSAPATGGVKme2KPHR selected as a 4+ precursor ion.
453.7857
QEx2_027265
#
3021
RT:
13.28
AV:
1
NL:
2.84E4
T:
FTMS + p NSI d Full ms2 384.2269@hcd30.00 [80.0000-1590.0000]
0
100
150
200
250
300
350
400
450
500
550
600
650
700
750
800
850
900
m/z
z=2
100
95
90
156.1023
85
z=1
80
75
359.1929
70
504.3097
z=1
84.0814
65
z=2
z=?
60
175.1195
55
z=1
Relative Abundance
50
45
539.8288
386.5685
40
z=3
z=2
409.2309
35
z=1
30
131.0817
z=1
341.1825
425.2753
25
z=1
239.0907
579.3496
z=2
z=1
20
z=2
588.3546
288.1552
z=1
693.4526
15
225.0426
z=2
495.8048
z=?
270.1447
z=1
295.1770
z=2
85.1018
z=?
10
z=?
z=?
599.3877
214.6291
732.4466
z=?
z=?
z=1
5
904.5315
675.4258
833.4979
z=?
z=?
z=?
QEx2_027264
#
3875
RT:
17.01
AV:
1
NL:
8.66E3
T:
FTMS + p NSI d Full ms2 384.2267@hcd27.00 [80.0000-1590.0000]
453.7855
z=2
100
100
150
200
250
300
350
400
450
500
550
600
650
700
m/z
95
90
85
239.0907
z=1
80
75
504.3088
70
z=2
65
60
55
Relative Abundance
50
156.1022
225.0435
84.0813
386.5692
45
175.1190
z=?
z=1
z=?
z=3
z=?
40
367.0708
z=?
539.8252
35
359.1933
392.5718
z=?
z=3
579.8514
z=2
30
152.1073
425.2743
355.1971
85.1018
z=?
295.1033
588.3556
z=?
z=?
25
z=?
z=2
z=2
409.2310
z=2
530.8233
117.0907
20
z=1
z=2
z=?
303.1948
273.0293
z=3
z=?
15
10
495.3033
330.5410
97.1014
z=?
221.0843
z=?
652.0468
z=?
z=?
5
z=?
0
NCE = 30%
NCE = 27%
QEx2_027267
#
3041
RT:
13.40
AV:
1
NL:
1.94E4
T:
FTMS + p NSI d Full ms2 384.2269@hcd36.00 [80.0000-1590.0000]
84.0814
z=1
100
100
150
200
250
300
350
400
450
500
550
600
650
700
750
m/z
95
90
85
156.1022
80
z=1
75
70
175.1194
65
z=1
60
359.1926
55
Relative Abundance
50
z=1
45
409.2311
40
453.7850
z=1
35
z=2
30
239.0905
z=1
25
131.0815
504.3083
z=?
242.1506
176.1225
341.1827
20
537.3244
z=?
z=1
z=1
z=1
85.1018
693.4512
285.2289
225.0429
z=2
15
z=?
366.7291
z=?
z=?
z=3
539.8296
270.1458
112.0872
425.2775
10
z=1
396.7665
z=2
z=?
z=?
216.0976
311.1700
z=2
z=?
z=?
z=1
471.2936
599.3873
732.4528
579.3516
5
z=?
z=?
z=?
z=?
0
QEx2_027266
#
2992
RT:
13.18
AV:
1
NL:
1.52E4
T:
FTMS + p NSI d Full ms2 384.2268@hcd33.00 [80.0000-1590.0000]
0
100
150
200
250
300
350
400
450
500
550
600
650
700
750
800
850
900
m/z
156.1024
z=1
100
95
453.7859
84.0814
90
z=1
z=2
85
175.1194
80
359.1930
z=1
75
z=1
70
65
60
55
Relative Abundance
50
504.3095
45
409.2317
z=1
z=2
40
239.0902
z=1
35
530.8248
z=2
30
131.0816
537.3268
z=?
341.1813
z=1
25
z=?
242.1505
225.0434
20
z=?
425.2740
z=?
z=2
288.1560
693.4506
386.5692
579.3450
85.1017
z=?
z=3
498.3410
15
z=2
z=?
z=?
z=1
588.3560
216.0988
732.4578
833.4962
10
z=?
z=?
z=1
z=?
302.8587
904.5355
z=?
5
z=?
NCE = 36%
NCE = 33%

## Slide 17
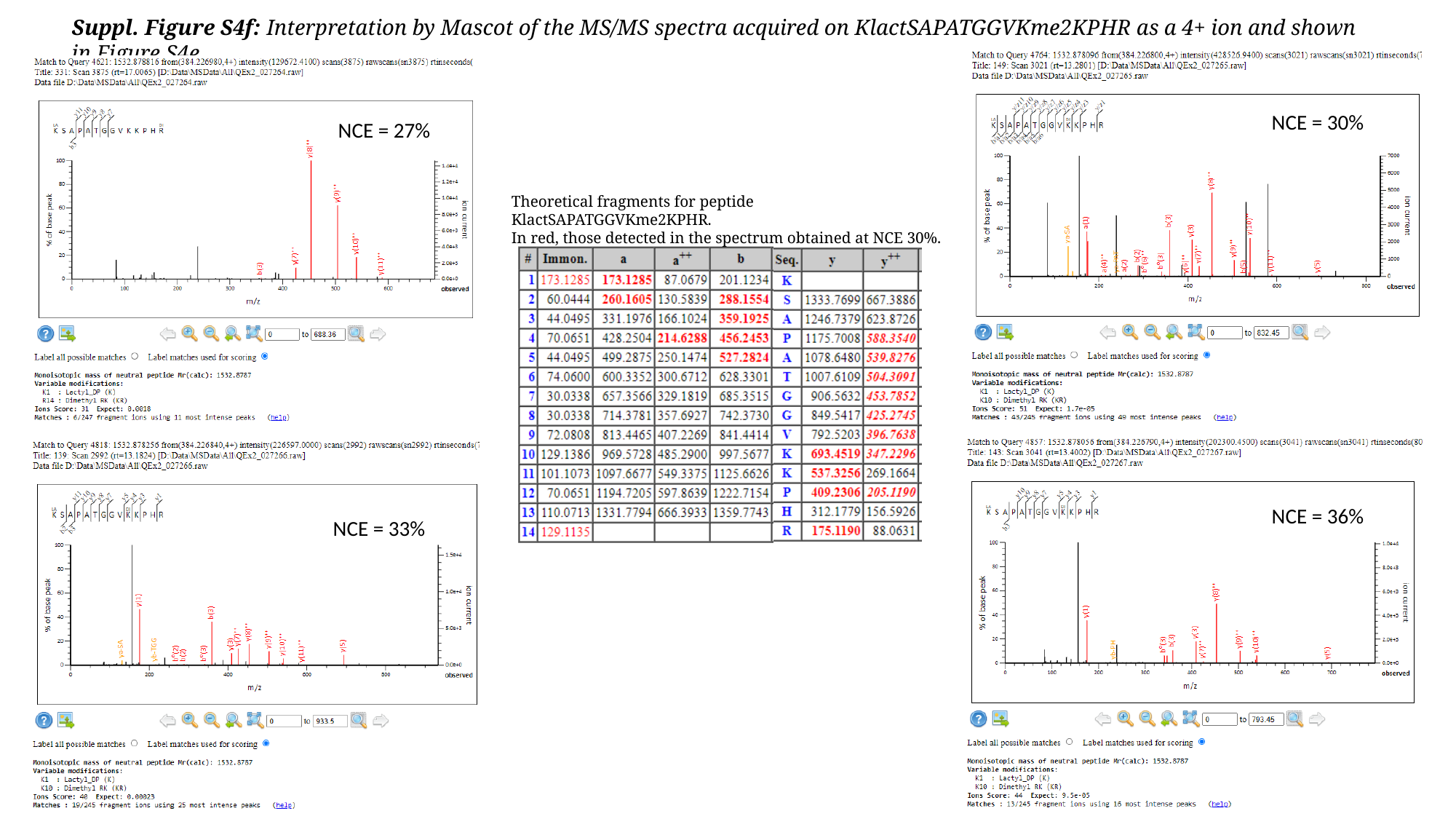

Suppl. Figure S4f: Interpretation by Mascot of the MS/MS spectra acquired on KlactSAPATGGVKme2KPHR as a 4+ ion and shown in Figure S4e.
NCE = 30%
NCE = 27%
Theoretical fragments for peptide KlactSAPATGGVKme2KPHR.
In red, those detected in the spectrum obtained at NCE 30%.
NCE = 36%
NCE = 33%

## Slide 18
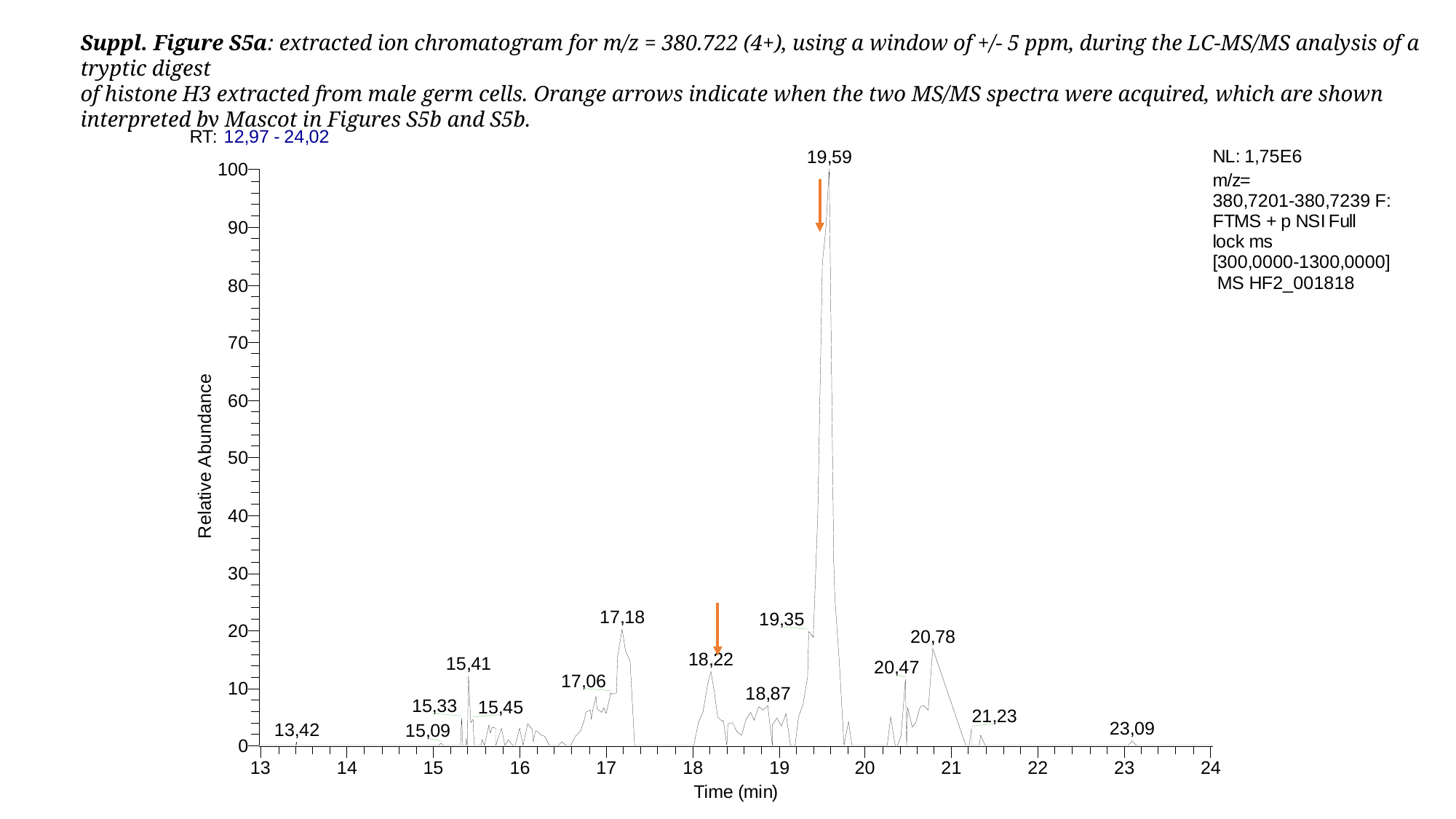

Suppl. Figure S5a: extracted ion chromatogram for m/z = 380.722 (4+), using a window of +/- 5 ppm, during the LC-MS/MS analysis of a tryptic digest
of histone H3 extracted from male germ cells. Orange arrows indicate when the two MS/MS spectra were acquired, which are shown interpreted by Mascot in Figures S5b and S5b.

## Slide 19
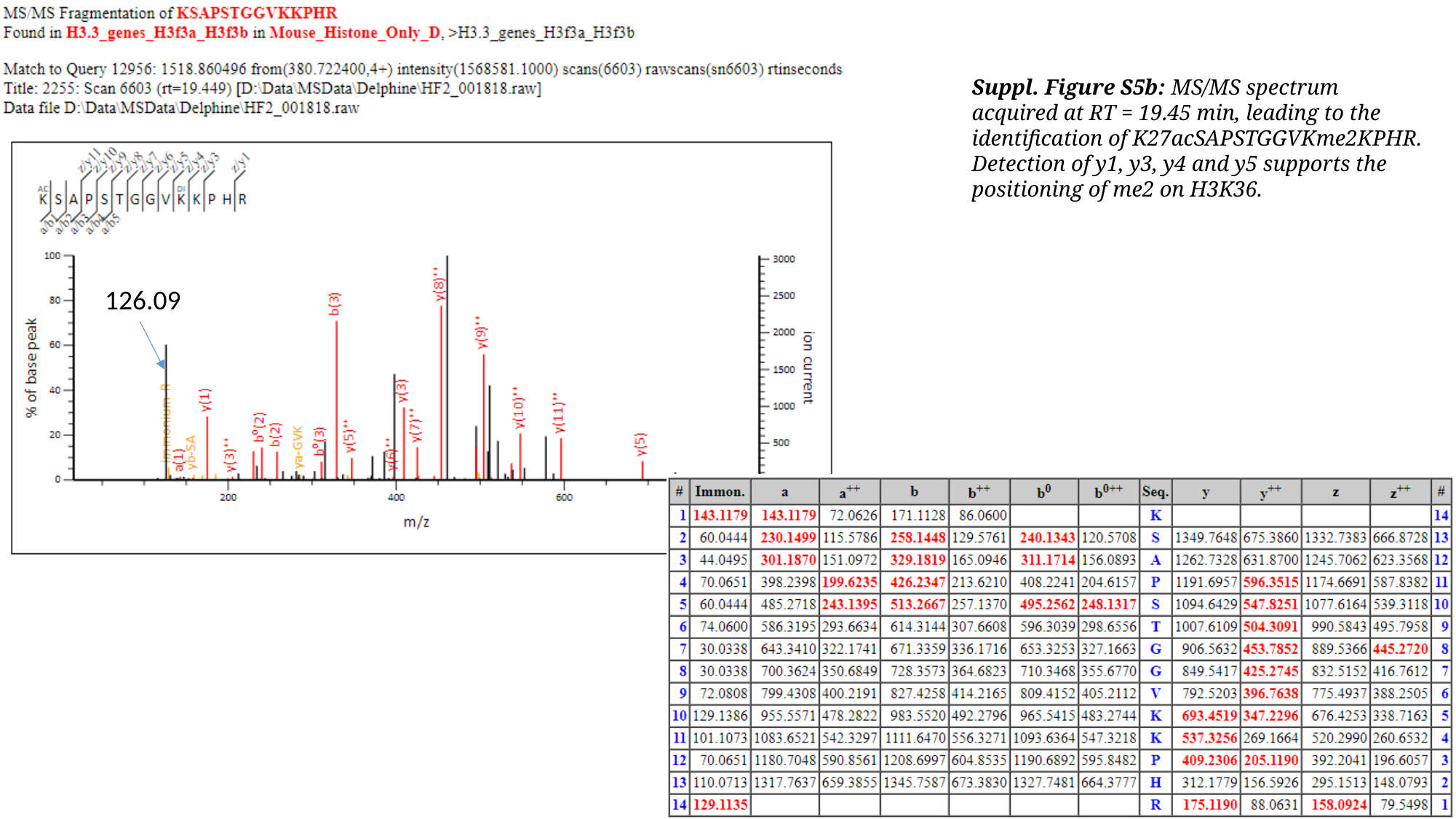

Suppl. Figure S5b: MS/MS spectrum acquired at RT = 19.45 min, leading to the identification of K27acSAPSTGGVKme2KPHR.
Detection of y1, y3, y4 and y5 supports the positioning of me2 on H3K36.
126.09

## Slide 20
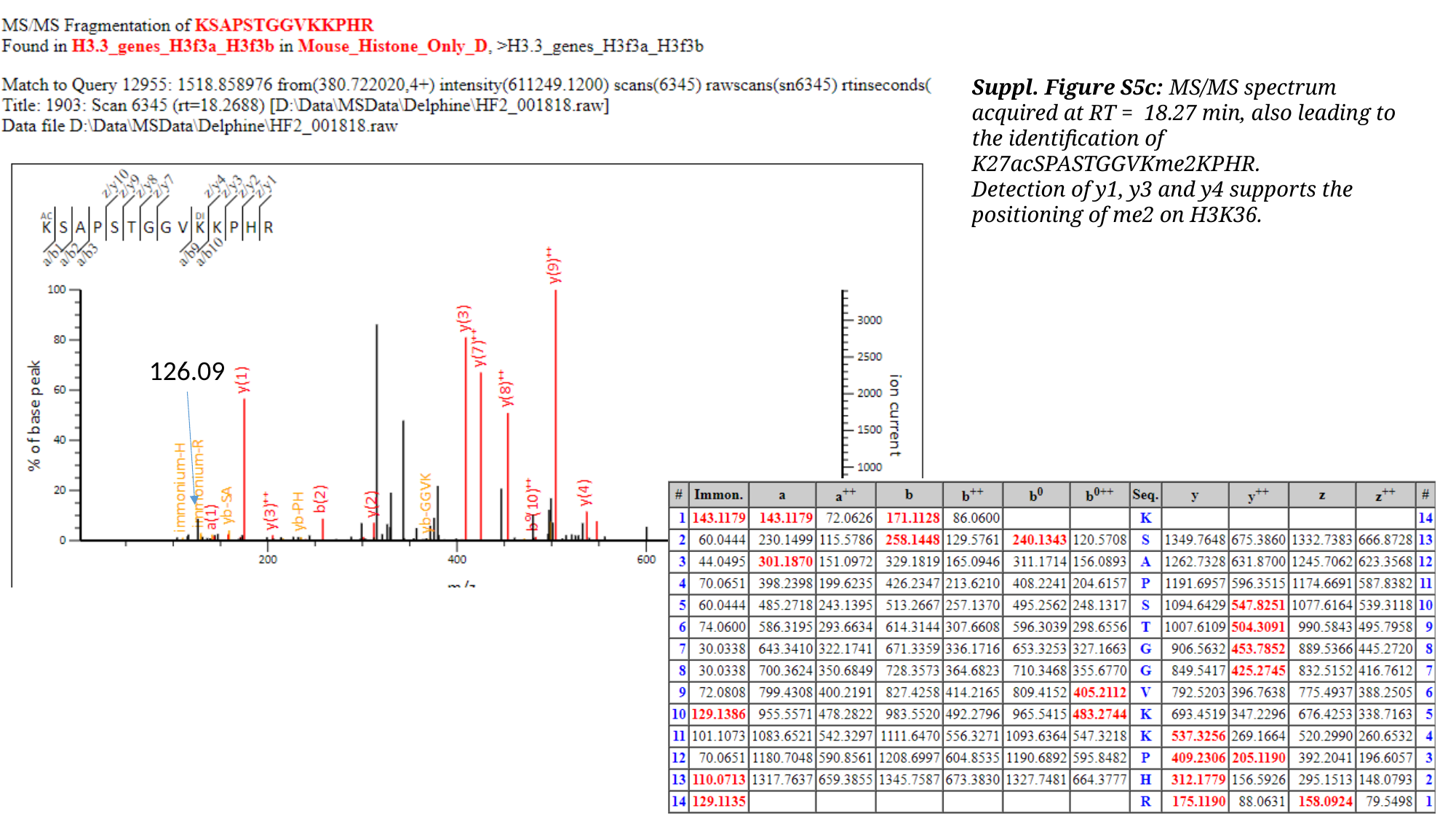

Suppl. Figure S5c: MS/MS spectrum acquired at RT = 18.27 min, also leading to the identification of K27acSPASTGGVKme2KPHR.
Detection of y1, y3 and y4 supports the positioning of me2 on H3K36.
126.09
